# Development and verification of lymphangiogenesis score for prediction of prognosis and immune landscape in gastric cancer

**DOI:** 10.3389/fimmu.2025.1595592

**Published:** 2025-11-04

**Authors:** Shihe Liu, Qiying Song, Runkai Chen, Di Wu, Xinxin Wang

**Affiliations:** Department of General Surgery, The First Medical Center of Chinese People's Liberation Army General Hospital, Beijing, China

**Keywords:** lymphangiogenesis, prognosis, immune cell infiltration, immunotherapy, gastric cancer

## Abstract

**Background:**

Gastric cancer (GC) is a leading gastrointestinal malignancy carrying a poor prognosis. Lymphangiogenesis (LYM) refers to the process of forming new lymphatic vessels. This process facilitates tumor metastasis and represents a promising therapeutic target in GC management. However, the exact mechanisms of LYM in GC remain incompletely understood.

**Method:**

The RNA-sequencing gene expression dataset and clinical characteristics of GC patients were obtained from The Cancer Genome Atlas (TCGA) and the Gene Expression Omnibus (GEO) database. The LASSO Cox regression method was utilized to identify feature genes and construct a Lymphangiogenesis Score (LYMS). A nomogram was constructed to assess the predictive efficacy of LYMS in the prognosis of GC patients. The gene set enrichment analysis (GSEA) employed to investigate different molecular functions and pathways. The immune microenvironment analysis, immunotherapy response analysis, and drug sensitivity were conducted to elucidate the association between LYMS and both immune landscape and immunotherapy response.

**Results:**

This study selected six LYM-related genes (ADAMTS1, SVEP1, CAV1, NOX4, NPTX1, and SPARC) to construct the LYMS. The results demonstrated that GC patients with a high LYMS exhibited significantly poorer prognosis. Distinct enrichment patterns of molecular functions and pathways were observed between the high and low LYMS groups. Furthermore, marked differences in immune landscape were identified. Immunotherapy response analysis and drug sensitivity analysis further indicated that high-LYMS patients showed reduced benefit to immunotherapy and diminished efficacy of certain chemotherapy agents.

**Conclusion:**

Overall, this study confirmed that LYMS is an independent prognostic risk factor in GC patients. The LYMS demonstrates significant predictive ability for responses to immunotherapy, suggesting its potential to guide future immunotherapy interventions for GC patients.

## 1 Introduction

Gastric cancer (GC) is the fifth prevalent malignancy in the world and the fourth most prevalent cause of cancer-associated deaths (1). In spite of continuous advancements in therapeutic strategies, GC patients prognosis remains suboptimal (2). Advancing insights into the mechanisms underlying GC have catalyzed the development of new therapeutic strategies, such as immune checkpoint inhibitors (ICIs), cellular immunotherapy, and cancer vaccines (3). GC is a kind of lymphatic metastatic tumor, characterized by the spread of cancer cells to adjacent lymph nodes via lymphatic vessels. This type of metastasis often occurs at an early stage and may affect multiple lymph nodes (4). Therefore, elucidating the potential mechanisms of GC and lymphatic metastatic is crucial for effective prevention and treatment.

The lymphatic system plays a critical role in collecting and transporting interstitial fluid, facilitating immune responses, and serving as a significant pathway for tumor metastasis (5). Lymphangiogenesis (LYM) refers to the formation of new lymphatic vessels, which is a crucial process for tumor cells to access the lymphatic system (6). Lymphangiogenesis is indispensable for pre-metastatic niche formation (7). Lymphatic vessels not only serve as a physical pathway for tumor cells but also facilitate their metastasis. Vascular endothelial growth factor (VEGF)-C and VEGF-D are critical factors in regulating lymphatic vessel development and growth (8, 9). Tumors secrete lymphangiogenesis factors, such as VEGF-C and VEGF-D, via lymphatic pathways, which promote the formation of lymphatic vessels and create favorable conditions for tumor metastasis, facilitating the spread and dissemination of cancer cells (10, 11). In GC, enhancing lymphangiogenesis and increasing lymphatic vessel permeability promotes lymphatic metastasis (12). Research has demonstrated that tumor-associated lymphangiogenesis is closely correlated with lymph node metastasis and poor clinical prognosis (13). And GC cells themselves can promote lymphangiogenesis by targeting the Akt/mTOR pathway to increase the protein expression of VEGF-C and VEGF-D (11). Additionally, lymphatic vessels not only function as pathways for the spread of tumor cells but also play a pivotal role in modulating the host immune response. Macrophages are crucial participants in lymphangiogenesis, as they secrete VEGF-C, VEGF-D, VEGFR3 and various inflammatory factors (14, 15). A wide variety of tumor-associated immune cells, including mast cells, macrophages, cancer-associated fibroblasts and lymphocytes, contribute to lymphangiogenesis through the secretion of pro-lymphangiogenic factors (16–18). LYM facilitates tumor metastasis and represents a promising therapeutic target in GC management. However, the exact mechanisms of LYM in GC remain incompletely understood.

In this study, we constructed LYMS signature and validated its predictive efficacy for the prognosis of GC patients by utilizing multiple databases and nomograms. Additionally, we investigated its correlation with the immune profile and assessed the effectiveness of immunotherapy through immune microenvironment analysis and immunotherapy response analysis. Furthermore, drug sensitivity analysis provides valuable references for the clinical treatment of GC patients. Our findings offer new evidence for the role of LYM in the progression of GC.

## 2 Materials and methods

### 2.1 Data collection

The genes related with LYM were obtained from Human Gene Database (GeneCards, https://www.genecards.org/) and relevant review articles (19). A total of 466 LYM-related genes were included in this study. The detailed list of these genes can be found in **Supplementary Table S1**.

The training dataset comprised transcriptomic profiles and corresponding clinical data for 412 GC patients and 36 control subjects, sourced from the TCGA- STAD database. For external validation, two independent cohorts were utilized: GSE84437 containing 433 GC patients and GSE84437 containing 357 GC patients, both retrieved from the Gene Expression Omnibus (GEO).

### 2.2 Differential expression analysis

A total of 466 LYM-related genes were subjected to differential expression analysis comparing 412 GC samples with 36 normal tissue samples from the TCGA cohort. The “limma”R package was used to identify differentially expressed genes (DEGs) associated with LYM, and genes with an absolute log-fold change (|logFC|) >1 and an adjusted p-value < 0.05 were classified as statistically significant DEGs (20).

### 2.3 Pathways and function enrichment analysis of DEGs

The R package “clusterProfiler” was utilized to conduct Gene Ontology (GO) and Kyoto Encyclopedia of Genes and Genomes (KEGG) enrichment analyses to evaluate the potential biological pathways associated with LYM-related DEGs (21). The GO analysis comprised three categories: Biological Processes (BP), Molecular Functions (MF), and Cellular Components (CC), with statistical significance thresholds set at an adjusted p-value < 0.05 and q-value < 0.05.

### 2.4 Development and validation of LYM score

Three independent cohorts comprising 385 GC patients from TCGA, 433 from GSE84437, and 357 from GSE84433 with available survival data were included in the analysis. Univariate Cox regression analysis was performed on the above three databases to identify genes with significant prognostic value (p < 0.05), and common genes were determined through intersection. The least absolute shrinkage and selection operator (LASSO) Cox regression method was employed to identify candidate genes, and the optimal signature was constructed using the “glmnet” package. Subsequently, the LYM score was calculated as follows: LYMS =∑ (βi Genei), where βi denotes the risk coefficient, and Genei represents the expression level of each gene. Patients were stratified into high- and low-LYMS groups based on median LYMS values. Kaplan Meier analysis was conducted using “survival” and “survminer” R packages to assess the association between LYMS and overall survival (OS). Finally, the LYMS was further validated in the GSE84437 and GSE84433 datasets.

### 2.5 Construction of a nomogram

An innovative prognostic nomogram was developed by integrating clinical characteristics, including age, TNM stages and LYMS through multivariate Cox and stepwise regression analyses. Calibration plots were used to evaluate the nomogram’s predictive accuracy. Additionally, time-dependent the receiver operating characteristic (ROC) curves, implemented via the R “timeroc” package, were utilized to assess the area under the curve (AUC) for the nomogram.

### 2.6 Gene set enrichment analysis

GSEA was conducted to identify differentially enriched pathways between GC patients with high-LYMS and low-LYMS. The “clusterProfiler” R package was utilized to conduct GSEA. In accordance with established GSEA guidelines, statistically significant results were defined as results meeting a significance threshold of p<0.05 and FDR<0.25 (22).

### 2.7 Immune cell landscape and immune microenvironment analysis

This study analyzed the association between the LYMS and immune cell infiltration utilizing various algorithms, including the CIBERSORT-ABS, EPIC, QUANTISEQ, TIMER, MCPCOUNTER, XCELL, and EPIC algorithms. Additionally, the stromal score, immune score, tumor purity, and ESTIMATE score were utilized to compare the tumor microenvironment (TME) between GC patients with high and low LYMS through the ESTIMATE algorithm (23).

### 2.8 Immunotherapy response analysis and drug sensitivity

Additionally, we employed the Tumor Immune Dysfunction and Exclusion (TIDE) algorithm (http://tide.dfci.harvard.edu/) to predict immunotherapy responses between LYMS groups (24). Tumor mutation burden (TMB) between high- and low-LYMS groups was compared using somatic mutation data analyzed with the “maftools” package. Immunophenoscore (IPS), obtained from The Cancer Immunome Atlas (TCIA, https://tcia.at/home), was used to assess responses to immunotherapy across different risk groups. To evaluate drug sensitivity, the half maximal inhibitory concentration (IC50) values were calculated using data of GC obtained from Genomics of Drug Sensitivity in Cancer, with predictions generated via the “oncoPredict” package (25).

### 2.9 Immunohistochemical (IHC) analysis

IHC analysis leverages antigen-antibody specificity binding to detect and localize target antigens in cellular and tissue contexts. We evaluated the expression of key genes in GC and normal tissues using IHC data from the Human Protein Atlas (HPA) database (http://www.proteinatlas.org/) (26).

### 2.10 Cell-line culture and quantitative real time PCR

The GES1, AGS, HGC27 and MKN1 cell lines were obtained from the General Surgery Laboratory of Chinese PLA General Hospital. The cell culture conditions were as follows: RPMI 1640 (G4535A, Servicebio, China) + 10% FBS (G8003, Servicebio, China) at 37 °C and 5% CO2. The steps of quantitative real-time PCR (qRTPCR) were as follows: Total RNA was isolated from the cells using FreeZol reagent (R711, Vazyme, China), and the extracted total RNA was reverse transcribed to cDNA using HiScript IV All-in-one Ultra RT SuperMix reagent (R433, Vazyme, China), and stained using SupRealQ Purple Universal SYBR qPCR Master mix (Q412, Vazyme, China) for staining, and the expression levels of the mRNAs of the 6 genes were detected according to the intensity of the fluorescent signals. β-actin mRNA expression level was used as an endogenous control. Three experiments were performed for each sample, and the results were used to calculate the expression values of the 4 genes according to Equation 2^-△△Ct^. The primer sequences are shown in **Supplementary Table S2**.

### 2.11 Statistical analysis

Statistical analyses were performed in R (v4.3.0) and Free Statistics software version 1.9.2. Group differences were analyzed using Student t-test or Wilcoxon test. Survival outcomes associated with categorical variables were assessed via Kaplan-Meier analysis. Statistically significant difference was defined as p<0.05.

## 3 Results

### 3.1 Differentially expressed genes analysis and enrichment analysis

**Figure 1:**
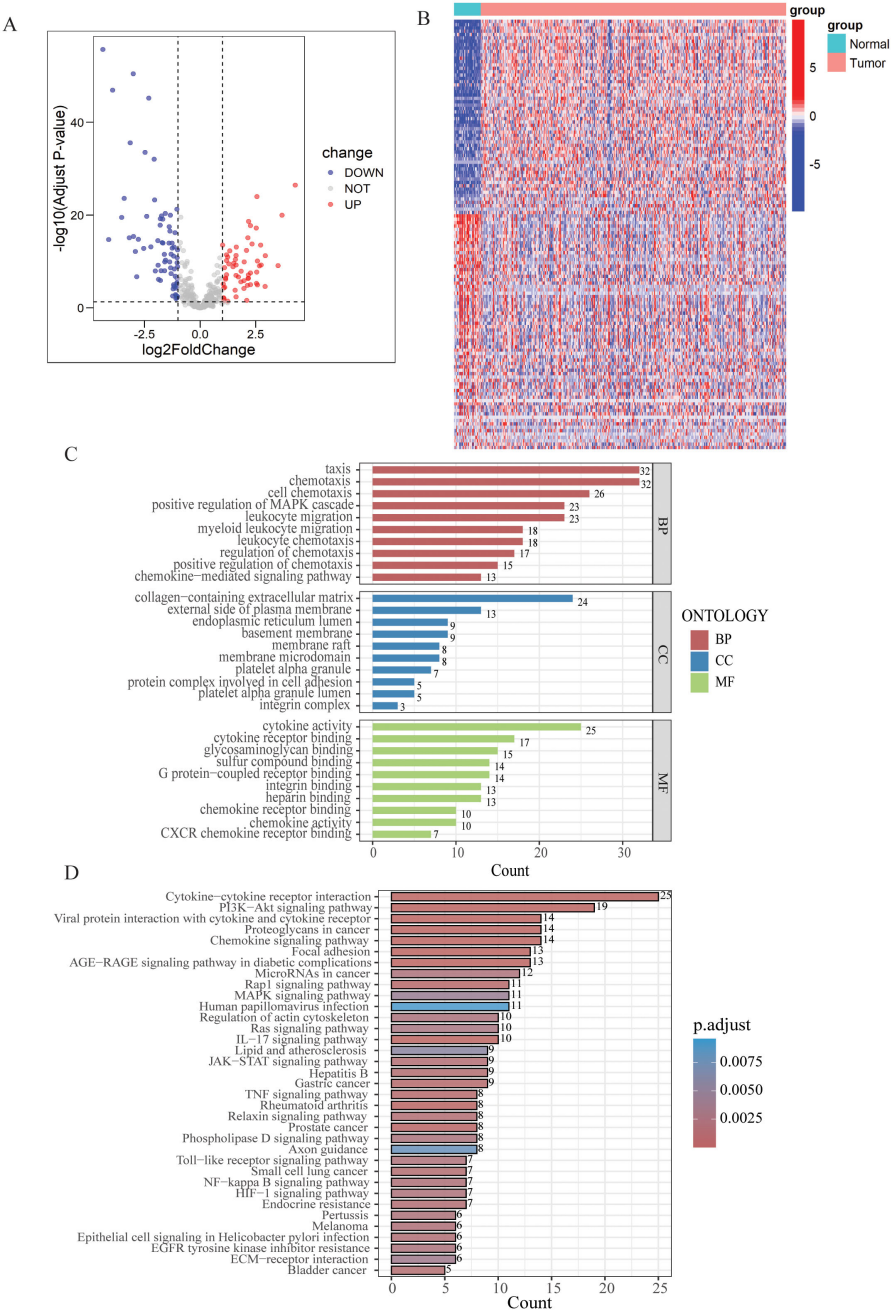
Differential expression analysis and enrichment of LYM-related genes. **(A)** Volcano map of the LYM-related DEGs between GC and normal tissues from TCGA cohort. **(B)** Heatmap of the LYM-related DEGs between GC and normal tissues from TCGA cohort. **(C)** GO enrichment analyses based on the DEGs. **(D)** KEGG enrichment analyses based on the DEGs.

In the TCGA-STAD cohort, 128 DEGs were identified between normal samples and gastric tumor samples using the limma analysis. This included 58 upregulated genes and 70 downregulated genes (**Figure 1A**, **Supplementary Table S3**). The expression patterns of DEGs were illustrated in the heatmap presented in **Figure 1B**.

Then, we conducted GO and KEGG enrichment analysis based on the 128 DEGs. GO analysis results displayed the top 10 results in BP, MF and CC (**Figure 1C**). Based on the results of CC, DEGs related to LYM were predominantly enriched in the collagen-containing extracellular matrix (ECM). Interactions between the ECM and lymphatics, as well as the biophysical characteristics of the stroma, influence tumor formation, growth, and metastasis (27). ECM stiffness stimulates the expression of globin transcription factor (GATA) binding protein 2 and GATA2-dependent VEGFR-3, mediating the growth and migration of lymphatic endothelial cell *in vivo (* 28). The results of the BP analysis indicated that the DEGs were closely associated to the chemotactic process, including cell chemotaxis, regulation of chemotaxis, and chemokine-mediated signaling pathway. The results of the MF analysis indicated that LYM was closely associated with cytokine activity. VEGFs are key cytokines involved in the LYM process, particularly VEGF-C and VEGF-D, which are known to be the main mediators of lymphatic endothelial cell proliferation and migration (29).

The results of KEGG analysis suggested that the LYM-related DEGs were significantly enriched in the following pathways: cytokine-cytokine receptor interaction, PI3K−AKT signaling pathway, proteoglycans in cancer, chemokine signaling pathway and focal adhesion (**Figure 1D**). These results provided novel insights in the mechanisms of LYM.

### 3.2 Constructing the LYM score

**Figure 2:**
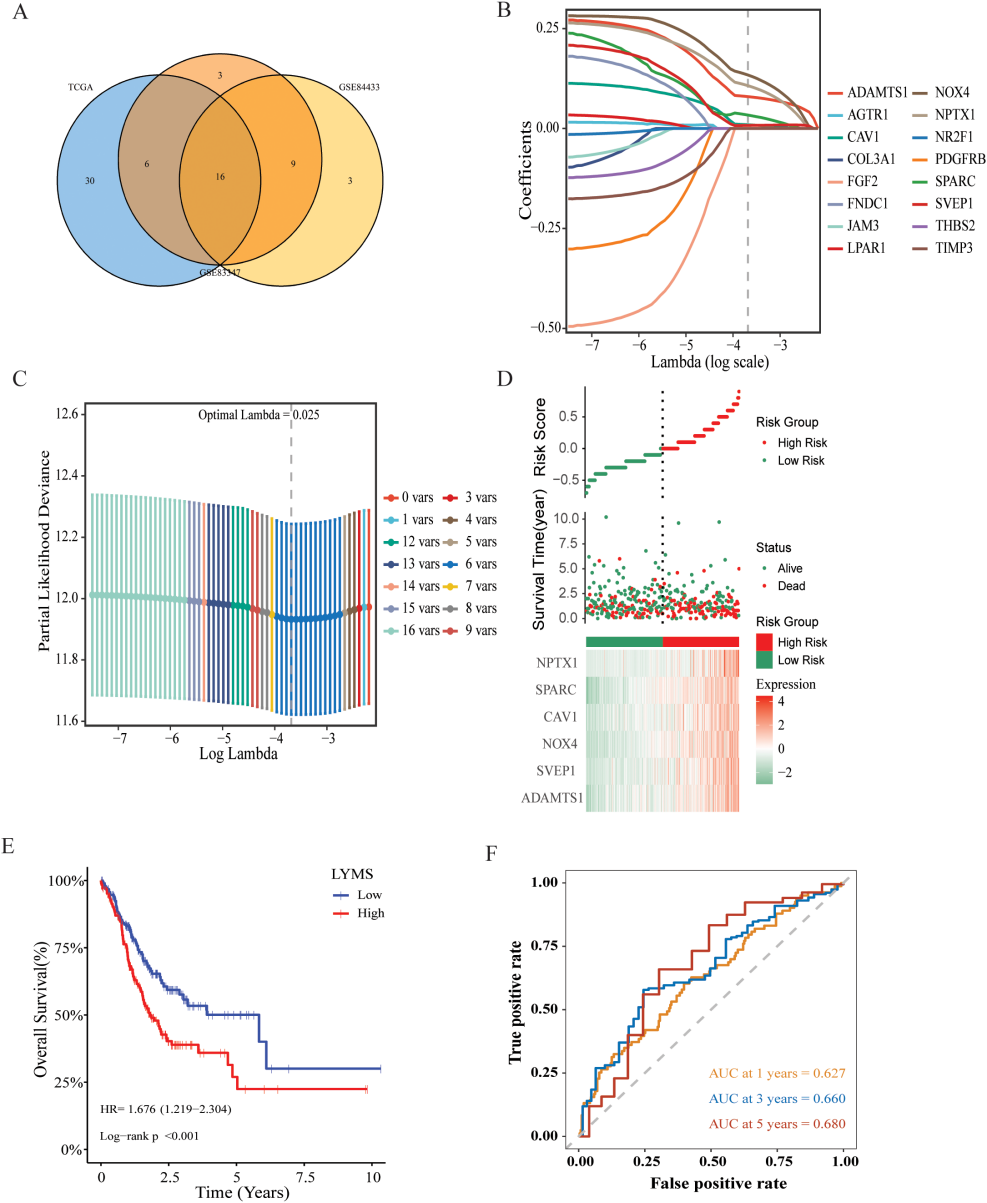
Construction and validation of a prognostic gene signature for GC patients. **(A)** Venn diagram representing common LYM-related DEGs with potential significant prognostic value across the three databases. **(B)** LASSO coefficient profiles of 16 common genes **(C)** Cross-validation of the constructed signature. **(D)** Distribution of risk score, survival status and heatmap of LYMS including 6 genes in TCGA cohort. **(E)** The KM analysis of LYMS predicting the OS of patients in TCGA cohort. **(F)** The ROC curves evaluating the predictive accuracy of LYMS at 1-,3- and 5-years in TCGA cohort. LYMS, lymphangiogenesis score; KM, Kaplan-Meier; AUCs, areas under the curve.

This study collected survival data from GC patients and performed further analyses. Univariate Cox regression analysis was employed to identify prognosis-associated genes across three gene databases. The analysis revealed significant associations between genes expression and OS in GC patients across three cohorts: 52 genes in TCGA (p < 0.05), 34 genes in GSE84437 (p < 0.05), and 28 genes in GSE84433 (p < 0.05). 16 genes were identified as common among TCGA, GSE84437, and GSE84433 cohorts (**Figure 2A**, **Supplementary Figure S1**). Then, LASSO-Cox regression analysis was conducted on these genes to select the optimal penalty parameter (lambda value λ =0.025). The results of this analysis identified six genes: a disintegrin and metalloprotease with thrombospondin motifs 1 (ADAMTS1), sushi, von Willebrand factor type A, EGF and pentraxin domain containing 1 (SVEP1), caveolin-1 (CAV1), NADPH oxidase 4 (NOX4), neuronal pentraxin 1 (NPTX1), and secreted protein acidic and rich in cysteine (SPARC) (**Figures 2B, C**). Kaplan-Meier analysis demonstrated that each model gene exhibited a significant association with OS in GC patients (p<0.05, **Supplementary Figure S2**), indicating that these genes served as risk factors (HR > 1, p < 0.05). Finally, regression coefficients were calculated for each of the 6 genes. A patient’s risk score was determined utilizing the following formula: LYMS = 0.0797 * ADAMTS1 + 0.0078 * SVEP1 + 0.1338 * NOX4 + 0.0095 * CAV1 + 0.0336 * SPARC + 0.1071 * NPTX1.

### 3.3 Validation of the LYMS model

According to the median LYMS (median=2.193), all GC patients in the TCGA database were divided into two groups: high-LYMS and low-LYMS. The distribution of risk scores, survival outcomes and differential expression of six signature genes between the two groups were visualized in **Figure 2D**. Kaplan-Meier survival analysis demonstrated a significantly poorer prognosis for high-LYMS patients compared to the low-LYMS group (p<0.001, **Figure 2E**). Time-dependent ROC curve analysis showed that the LYMS predicted OS with AUC values of 0.627 (1-year), 0.660 (3-years), and 0.680 (5-years) (**Figure 2F**).

To validate the LYMS constructed from the TCGA cohort, patients in the GSE84437 and GSE84433 were also classified into high-LYMS and low-LYMS groups according to the median LYMS. Consistent with the training cohort, the high-LYMS group exhibited a significantly poorer prognosis than the low-LYMS group in both validation cohorts (p<0.001) (**Supplementary Figures S3A, B**). The distribution of risk scores and survival outcomes for samples in the validation cohort were illustrated in **Supplementary Figures S3C, D**, along with the differential expression of six genes in two groups. Additionally, the time-dependent ROC curves were presented in **Supplementary Figures S3E, F**.

VEGFs are key cytokines involved in the LYM process, particularly VEGF-C and VEGF-D, which are known to be the main mediators of lymphatic endothelial cell proliferation and migration (29). Further elucidating the relationship between LYMS and VEGFs helps to better understand LYMS. We analyzed the correlation between LYMS and VEGFs (**Supplementary Figures S4A–E**). The results revealed that LYMS exhibited positive correlations with VEGFB, VEGFC and VEGFD, but a negative correlation with VEGFA.

### 3.4 Development of a nomogram based on LYMS

This study explored the association of the LYMS with clinical futures, including the T, M, TNM stage and OS in GC patients. The results were presented in **Supplementary Figure S5**. Patients with poor prognosis, advanced T stage, and high TNM stage exhibited higher LYM scores.

**Figure 3:**
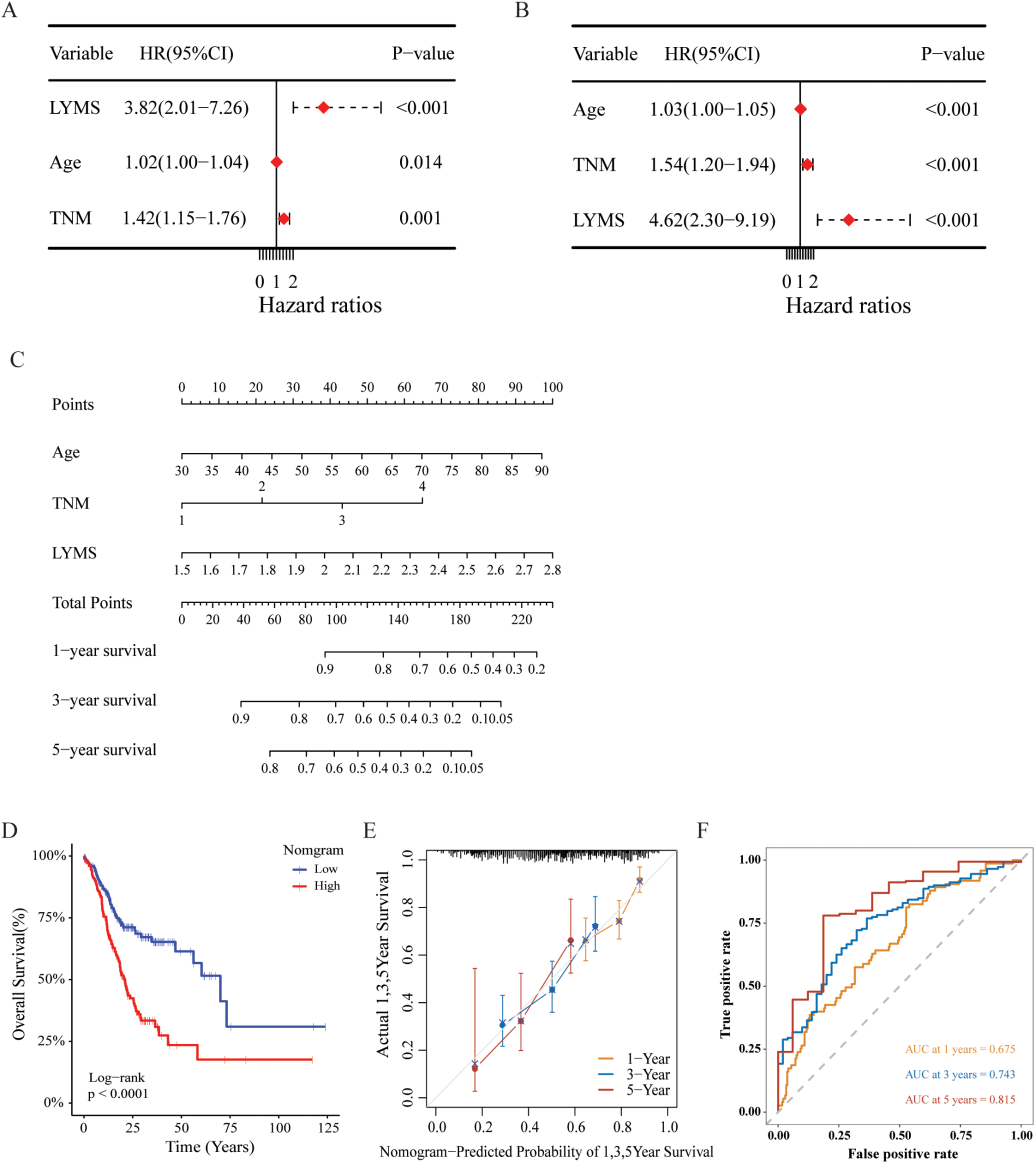
Construction and assessment of the nomogram survival model based on LYMS. **(A, B)** The univariate and multivariate analysis of LYMS and the clinical characteristics. **(C)** A nomogram was established based LYMS and clinical characteristics. **(D)** Kaplan-Meier analyses for the two groups based on the nomogram in TCGA cohort. **(E)** The calibration curve of the nomogram in TCGA cohort. **(F)** The ROC curves at 1-, 3-, and 5-years of nomogram in TCGA cohort. LYMS, lymphangiogenesis score; AUCs, areas under the curves.

We further investigated the prognostic significance of LYMS in GC patients. Univariate Cox regression analysis demonstrated that LYMS served as a significant risk factor (HR = 3.82, 95% CI 2.01-7.26, p<0.001, **Figure 3A**). Multivariate Cox regression analysis further identified LYMS as an independent prognostic risk factor (HR = 4.62, 95% CI 2.30-9.19, p<0.001, **Figure 3B**). Next, we incorporated factors such as LYMS, age, and TNM stage to develop a nomogram for the TCGA cohort. We employed multivariate Cox and stepwise regression analysis to predict the OS of GC patients (**Figure 3C**). The total points were negatively correlated with the survival rates of patients. Kaplan-Meier survival analysis indicated that GC patients with high total points on the nomogram had a worse prognosis compared to those with a low point (p<0.001, **Figure 3D**). The calibration curves demonstrated the nomogram’s robust predictive performance for OS at 1, 3, and 5 years (**Figure 3E**). Additionally, when predicting 1, 3-, and 5-years OS in GC patients, the nomogram demonstrated strong ability with AUC values of 0.675, 0.743, and 0.815, respectively (**Figures 3F**). The nomogram showed superior predictive accuracy for OS compared to individual parameters, including age, TNM stage, and LYMS alone (**Supplementary Figure S6**). Finally, external validation using the GSE84437 and GSE84433 databases confirmed the nomogram’s robust predictive performance (**Supplementary Figure S7**). These findings suggest the nomogram holds significant potential for clinical prognostication in GC, particularly for long-term (5-year) survival outcomes. Clinically, LYMS can predict the prognosis of gastric cancer and serves as an independent risk factor.

### 3.5 Immunological features of LYMS

**Figure 4:**
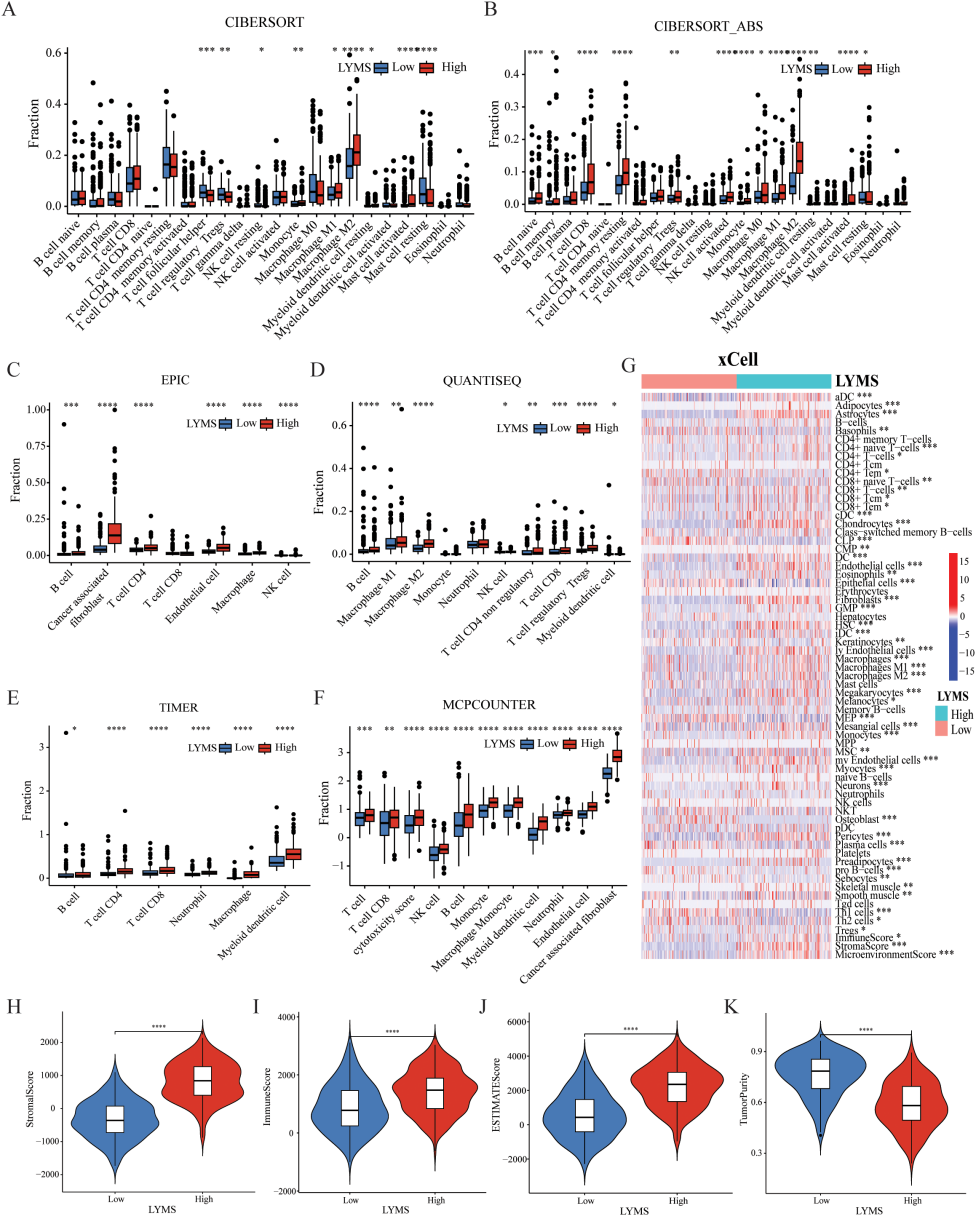
The correlations between LYMS and immune landscape. **(A-F)** Immune cell infiltration between Low-LYMS and High-LYMS groups based on CIBERSORT, CIBERSORT_ABS, EPIC, quanTIseq, TIMER and MCP-counter algorithms. **(G)** Correlation of LYMS with immune cell infiltration based on xCell algorithms. **(H-K)** The difference in tumor microenvironment between two groups based on Estimate algorithms. LYMS, lymphangiogenesis score. *p<0.05; **p<0.01; ***p<0.001; ****p<0.0001.

To investigate the association between LYMS and tumor immune features, we examined the immune cell infiltration within the TME. Given the complexity of the TME, we conducted comprehensive analyses including tumor-associated stroma content (Stromalscore), immune cell infiltration levels (Immunescore), tumor purity and the overall characteristics of the tumor microenvironment (Estimatescore) to ensure rigorous and multidimensional evaluation. Compared with CIBERSORT (**Figure 4A**), the results of (**Figure 4B**), EPIC (**Figure 4C**), QUANTISEQ (**Figure 4D**), TIMER (**Figure 4E**), MCPCOUNTER (**Figure 4F**), and XCELL (**Figure 4G**) indicated that the high LYMS group exhibited a higher proportion of B cells, CD8+ T cells, natural killer (NK) cells, macrophages, and other immune cells. However, the results of CIBERSORT (**Figure 4A**) indicated no significant difference or even presented contrary results in T cells and NK cells between two groups. The observed phenomenon may be attributed to the elevated stromal cell abundance within in the TME of the high LYMS group, including fibroblasts, endothelial cells, and matrix components, as demonstrated in **Figure 4G**. An increased stromal cell proportion could diminish the relative representation of specific immune cells. TME analysis further demonstrated reduced tumor purity scores in the high-LYMS group, whereas stromal, immune, and estimate scores were significantly elevated (**Figures 4H–K**).

### 3.6 Gene set enrichment analysis of LYMS

**Figure 5:**
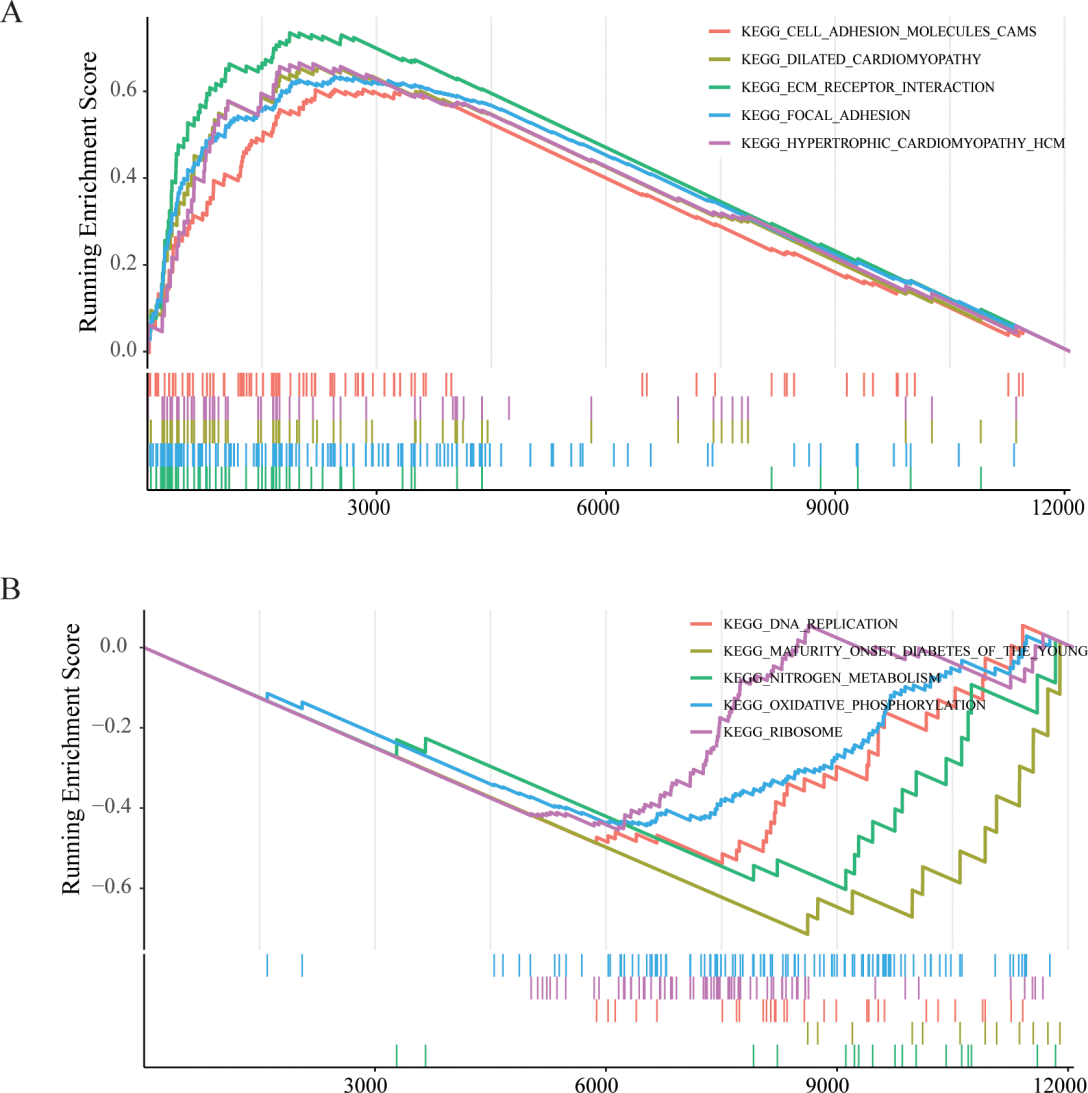
GSEA enrichment analyses between high LYMS group and low LYMS group. **(A)** Upregulated GSEA pathways in the high LYMS. **(B)** Downregulated GSEA pathways in the high LYMS. GSEA, Gene set enrichment analysis; LYMS, lymphangiogenesis score.

To further elucidate functional differences of LYMS, this article performed GSEA on patients with GC. The GSEA results indicated that cell adhesion molecules (CAMs), ECM receptor interactions, and focal adhesion were significantly activated in the high LYMS group (**Figure 5A**). In contrast, DNA replication, nitrogen metabolism, oxidative phosphorylation, and ribosome were inhibited in the high LYMS group (**Figure 5B**). These differential pathways may suggest the potential mechanisms underlying differences between two LYMS groups.

### 3.7 The correlation between LYMS and immunotherapy

**Figure 6:**
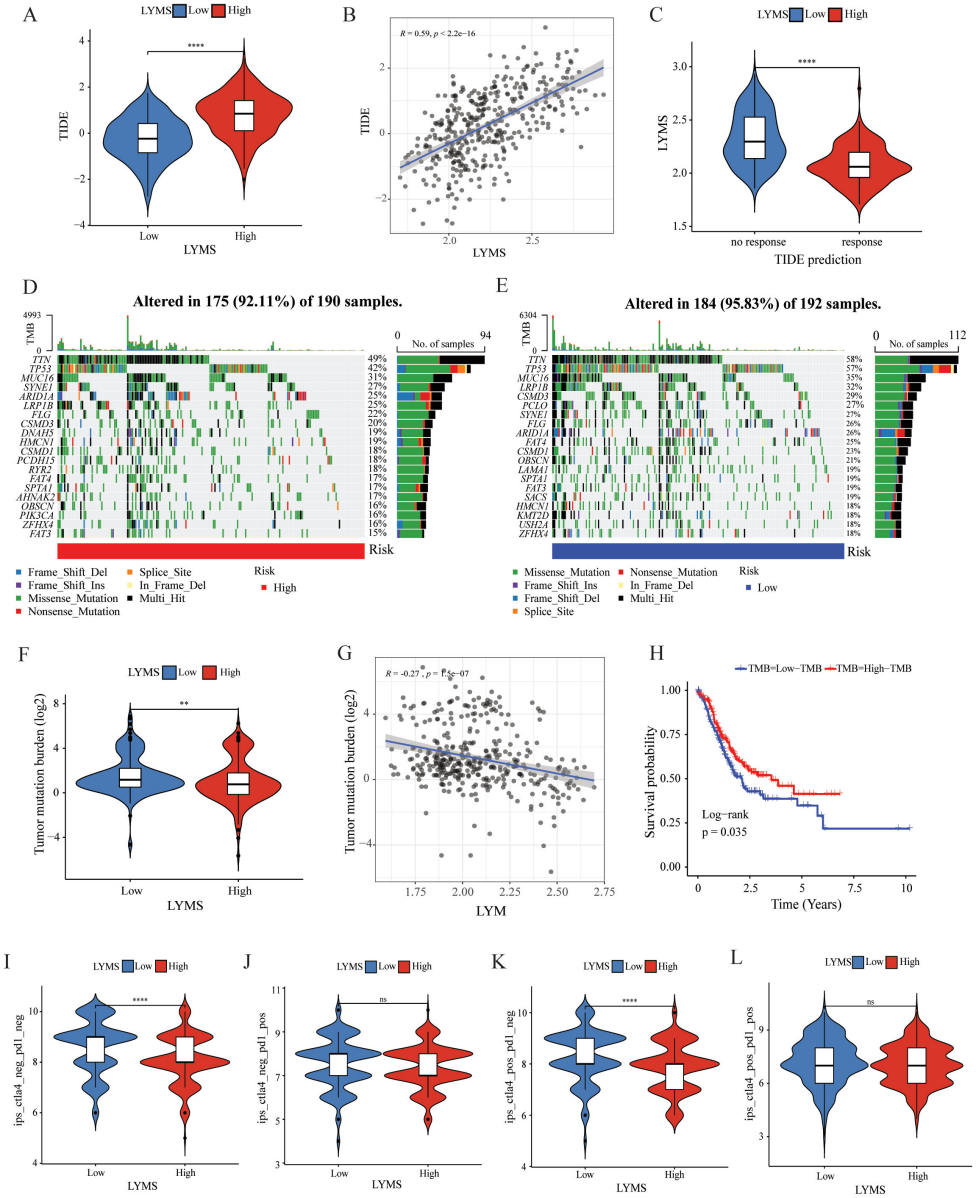
The correlation of LYMS with immunotherapy efficacy. **(A)** The difference in TIDE scores between high and low LYMS groups. **(B)** The correlation between TIDE Score and LYMS. **(C)** The difference in LYMS between the Responders and Non-responders. **(D)** TMB in the high LYMS group. **(E)** TMB in the low LYMS group. **(F)** The difference in TMB between high and low LYMS groups. **(G)** The correlation between TMB and LYMS. **(H)** The KM analysis in low-TMB and high-TMB groups. **(I–L)** The differences of Immunophenoscores (IPS) in four subgroups. TIDE, Tumor Immune Dysfunction and Exclusion; TMB, tumor mutational burden; LYM, lymphangiogenesis. **p<0.01; ****p<0.0001.

To further explore the role of LYMS in guiding GC immunotherapy, we performed correlation analyses between LYMS and three immunotherapeutic-related biomarkers: TIDE score, TMB, and IPS. TIDE results demonstrated that patients with elevated LYMS exhibited higher TIDE score (**Figures 6A, B**). High TIDE scores generally indicate that tumors possess robust immune evasion mechanisms, which suggests that patients with high LYMS may experience limited benefits from immunotherapy. To further elucidate the role of LYMS in predicting immunotherapy responsiveness, we conducted TIDE predictive analysis. The results demonstrated that GC patients who were responsive to therapy showed lower LYMS levels than non-responders (**Figure 6C**). Collectively, these findings indicated that GC patients with low-LYMS derived enhanced clinical benefits from immunotherapy relative to high-LYMS patients, which was in accordance with the TIDE results.

Subsequently, we examined the differences in TMB between two groups. Analysis indicated that the low LYMS group exhibited higher mutation frequency, including TTN, TP53, and LRP1B (**Figures 6D, E**). The TMB in the low LYMS group was significantly higher than that in the high LYMS group (**Figure 6F**). TMB exhibited a significant negative correlation with LYMS, with a correlation coefficient of -0.27 (**Figure 6G**). Furthermore, survival analysis further demonstrated that patients with high TMB exhibited a markedly improved prognosis compared to those with low TMB (**Figure 6H**). Finally, this study assessed the IPS of GC patients across different risk subgroups (**Figures 6I-L**, **Supplementary Figure S8**). Higher IPS score correlate with stronger sample immunogenicity (30). The results indicated that in the cytotoxic T-lymphocyte-associated protein 4 (CTLA4) -positive and programmed death receptor 1 (PD1) -negative subgroup, the low-LYMS group exhibited significantly elevated IPS scores compared to the high-LYMS group. Clinically, these findings indicated that GC patients with low-LYMS might derive enhanced clinical benefits from ICIs.

### 3.8 Drug sensitivity analysis

**Figure 7:**
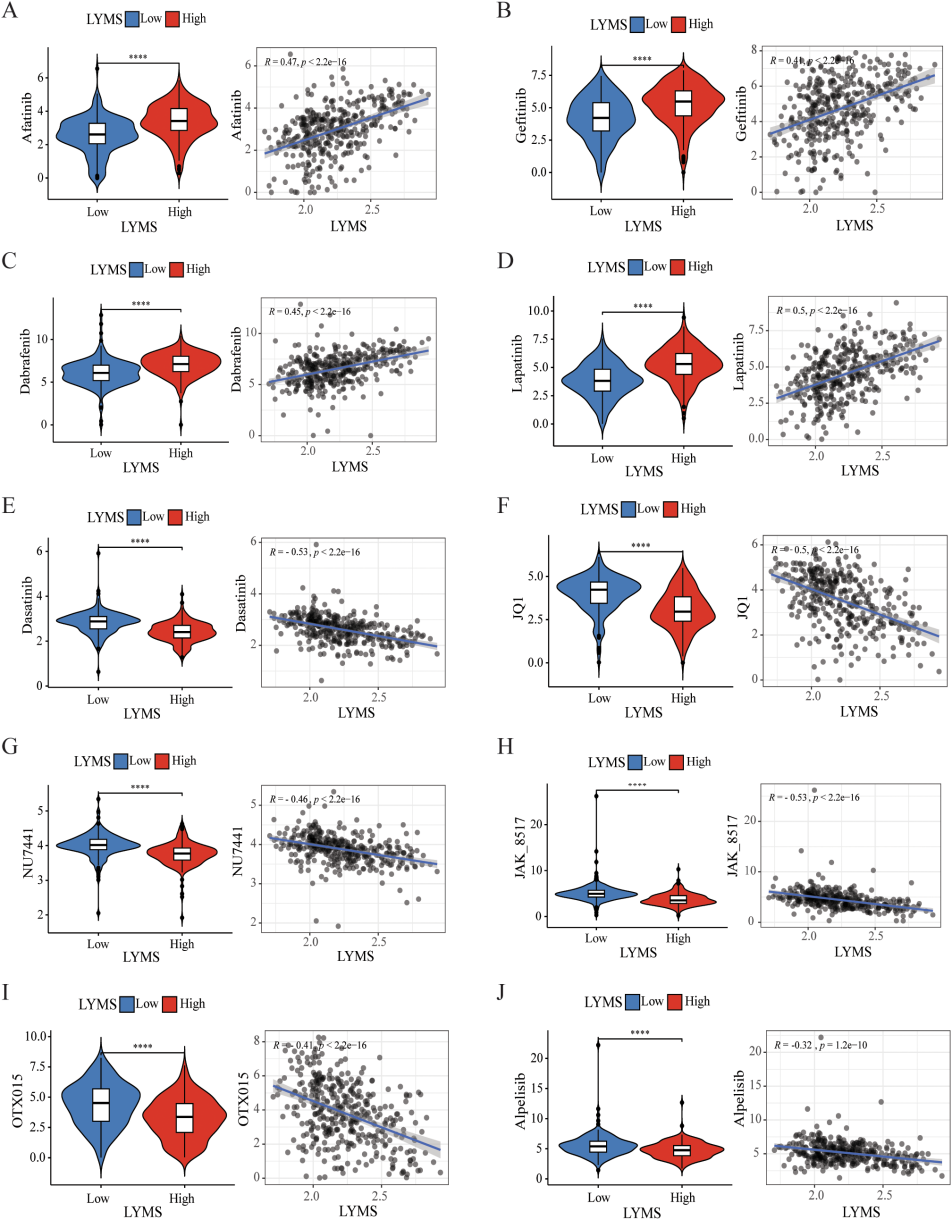
Efficacy of LYMS in predicting drug sensitivity. **(A-J)** The comparison of IC50 of drugs between high and low LYMS groups, and correlation between the IC50 and LYMS in GC patients. **(A)** Afatinib, **(B)** Gefitiinib, **(C)** Dabrafenib, **(D)** Lapatinib, **(E)** Dasatinib, **(F)** JQ1, **(G)** NU7441, **(H)** JAK8517, **(I)** OTX015, **(J)** Alpelisib. LYM, lymphangiogenesis. ****p<0.0001.

To investigate the correlation between LYMS and sensitivity to antitumor drugs in GC, we conducted a drug sensitivity analysis. The results indicated that the IC50 values of common drugs including afatinib, gefitinib, dabrafenib, and lapatinib (**Figures 7A–D**) were positively correlated with LYMS. This indicated that patients with high LYMS exhibited insensitivity to these drugs. Conversely, dasatinib, JQ1, NU7441, JAK8517, OTX015 and alpelisib (**Figures 7E–J**) demonstrated a negative correlation with LYMS, indicating that patients with high LYMS were more sensitive to these antitumor drugs, providing references for clinical drug selection.

### 3.9 Validation of the expression of featured genes

**Figure 8:**
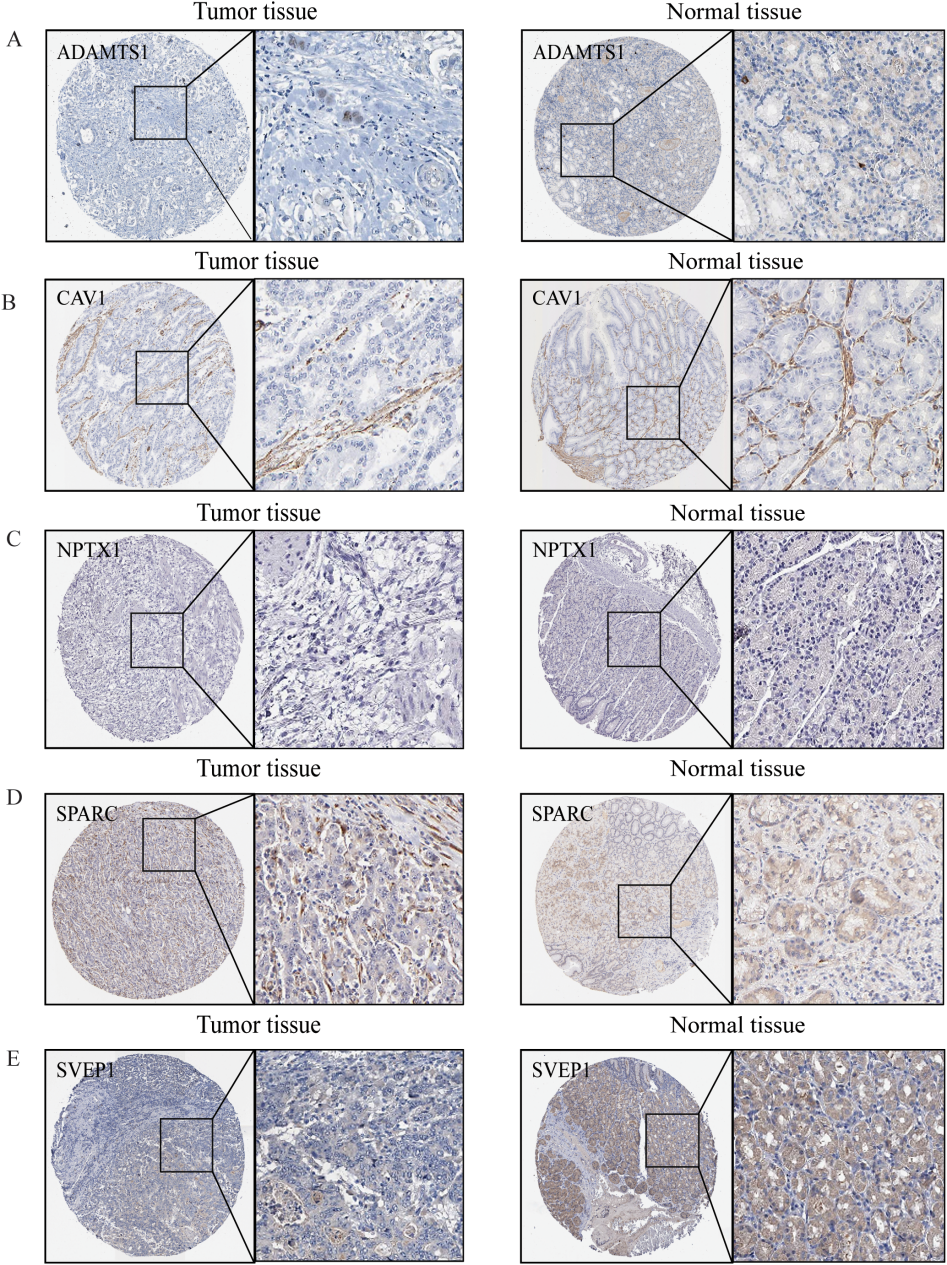
The protein expression levels of model genes. **(A)** ADAMTS1, **(B)** CAV1, **(C)** NPTX1, **(D)** SPARC, **(E)** SVEP1 in gastric normal tissues and gastric cancers from HPA online database.

IHC was employed to validate the expression of LYMS model genes in GC tissues. Compared to normal tissues, ADAMTS1 and SVEP1 expression levels were significantly downregulated in GC tissues, whereas SPARC expression was markedly upregulated (**Figure 8**). There were no significant differences for CAV1 and NPTX1. Additionally, HPA database lacks information on NOX4 expression in GC. Nevertheless, previous studies have reported significant upregulation of NOX4 in GC tissues compared to normal counterparts (31).

The qRT-PCR analysis revealed distinct expression profiles of six signature genes across gastric cancer cell lines (**Supplementary Figure S9**). ADAMTS1 demonstrated significantly higher expression levels in HGC27 cells (**Supplementary Figure S9A**). Both NPTX1 and SPARC exhibited consistently low expression across all three cell lines (HGC27, AGS, and MKN1) (**Supplementary Figures S9B, C**). While CAV1 showed elevated expression in HGC27, its expression was markedly reduced in MKN1 (**Supplementary Figure S9D**). Notably, NOX4 displayed substantial upregulation in all tested cell lines (HGC27, AGS, and MKN1) (**Supplementary Figure S9E**). SVEP1 elevated expression was observed in HGC27 and MKN1 (**Supplementary Figure S9F**). The differential expression patterns of these six signature genes between gastric cancer cells and normal gastric mucosal cells further validate the feasibility of the LYMS.

## 4 Discussion

Lymphangiogenesis is a crucial process that enables tumor cells to infiltrate the lymphatic system and plays a key role in tumor metastasis. However, there are limited clinical researches focused on the clinical characteristics, treatment, and prognosis of LYM in GC patients. In this study, we developed a LYMS model (ADAMTS1, SVEP1, CAV1, NOX4, NPTX1, and SPARC) using data from the TCGA cohorts and validated its robust efficacy in the GSE84437 and GSE84433 cohorts. Subsequently, patients were divided into two groups according to the median of LYMS. A nomogram model that integrated clinical futures with LYMS was established, demonstrating strong predictive performance for OS in GC patients. We further investigated the impact of LYMS on the tumor microenvironment and elucidated the relationship between LYMS and the response to immunotherapy, and sensitivity to chemotherapy drugs. Our findings indicated that patients with high LYMS might not benefit from immunotherapy; however, they exhibited higher sensitivity to drugs such as dasatinib and alpelisib. Clinically, LYMS can predict the prognosis of gastric cancer and serves as an independent risk factor. It assists in determining the benefits of immunotherapy, with patients in the high-LYMS group deriving limited benefits from ICIs. Additionally, it indicates drug sensitivity, providing references for clinical drug selection (e.g., dasatinib). In summary, LYMS enhances our understanding of the mechanisms underlying GC metastasis and progression, holding significant potential for prognostic prediction and guiding treatment decisions for GC patients.

ADAMTS1, a member of the matrix metalloproteinase family, is recognized for its role in inhibiting angiogenesis (32). In GC, elevated expression levels of ADAMTS1 were significantly linked to lymph node metastasis in primary tumors (33). Chien et al. reported that the activation of epidermal growth factor receptor mediated by the ADAMTS1/L1 cell adhesion molecule axis promoted the progression of epithelial-mesenchymal transition (EMT), thereby enhancing the invasive abilities of cancer cells (34). SVEP1 is a large ECM protein that plays a key role in regulating intercellular adhesion (35) and embryonic lymphatic development (36). SVEP1 has been shown to exhibit a significant association with poor prognosis in GC patients (37). Chen et al. found that abnormal expression of SVEP1 could promote tumor cell migration, chemotaxis, invasion, and proliferation (38). NOX4, a substrate of NADPH, is significantly upregulated in GC tissues and functions as a standalone indicator of unfavorable outcomes (31). Gao et al. revealed that NOX4 promoted GC metastasis by inducing EMT through the downstream JAK/STAT signaling pathway (39). Additionally, Nox4 has been shown to promote LYM via reactive oxygen species (ROS)/extracellular regulated protein kinases (ERK)/CCL21 pathway (40). The abnormal expression of NPTX1 promotes the invasion and proliferation of GC cells (41). Peng et al. discovered that NPTX1 suppressed cancer cell proliferation by regulating the retinoblastoma protein-E2F transcription factor signaling pathway via repression of cyclin A2 and cyclin-dependent kinases 2 (CDK2) expression (42). Moreover, NPTX1 has been shown to enhance chemotherapy sensitivity (43). SPARC, part of the ECM glycoprotein family, is significantly expressed in GC and correlates with depth of tumor invasion, lymph node metastasis, TNM staging (44), and poor OS (45). Huang et al. revealed that the LCN2/24p3R/JNK/c-Jun/SPARC axis drived GC malignant progression (46). CAV1, an oncogenic membrane protein linked to extracellular matrix organization, cell migration, and signaling, contributes to peritoneal metastasis in GC via the ROCK1/CAV1/Rab11 axis (47). Additionally, CAV1 is implicated in chemotherapy resistance in GC (48).

LYM contributes to gastric cancer progression and prognosis. Specifically, peritumoral lymphovascular density plays a critical role in lymph node metastasis, while intratumoral lymphovascular density is more strongly associated with tumor invasion depth (49). VEGFs are key cytokines involved in the LYM process, particularly VEGF-C and VEGF-D, which are known to be the main mediators of lymphatic endothelial cell proliferation and migration (29). Our results revealed that LYMS exhibited positive correlations with VEGFC and VEGFD. The LYMS genes are functionally connected to the VEGFs. SPARC regulates the expression of VEGF-C and VEGF-D in ovarian cancer, thereby affecting angiogenesis and lymphangiogenesis (50). Conversely, VEGF can also induce the expression of SPARC (51). The interaction between VEGF and SPARC jointly affects lymphangiogenesis. ADAMTS1 inhibits lymphangiogenesis by attenuating phosphorylation of the lymphatic endothelial cell-specific VEGF receptor (32). Conversely, VEGF significantly induces ADAMTS1 expression in endothelial cells in a protein kinase C-dependent way (52). CAV-1 regulates the expression of downstream VEGFs. Studies have shown that CAV-1 downregulation reduces insulin like growth factor-1-induced VEGFA secretion (53). CAV-1 modulates VEGF-stimulated VEGFR2 autophosphorylation and downstream angiogenic signaling (54). Additionally, the NOX4/ROS/VEGF pathway is involved in the regulation of VEGF expression (55). However, SVEP1, as a binding ligand of Tie1, affects specific aspects of lymphatic development in a VEGFC-independent manner (56).

Pathway enrichment analysis of 128 DEGs related to LYM between normal tissues and gastric tumor samples identified significant enrichment in the PI3K/AKT signaling pathway, Proteoglycan in cancer, RAP1(ras-related protein) signaling pathway and MAPK signaling pathway. Notably, PI3K/AKT inhibition suppresses EMT and LYM, thereby attenuating tumor invasion and metastasis (57). Proteoglycans exhibit diverse roles in tumor -associated LYM. Syndecan-4, a key lymphatic proteoglycan, acts as a key co-receptor for VEGF-C-mediated pathological LYM (58). In contrast, decorin interactes with VEGFR3 to suppress lymphatic vessel sprouting (59). RAP1 maintains lymphatic permeability, drives normal lymphatic development, and is essential for embryonic LYM and the maintenance of lymphatic junctions in adulthood (60). MAPK activation promotes LYM via the ERK/NF-κB pathway, increasing lymphatic vessel permeability and migratory capacity (61). Collectively, LYM is a multifactorial process regulated by multiple signaling pathways, and our findings elucidate novel mechanistic aspects of LYM.

GSEA revealed significant enrichment of cancer-related pathways in the high-LYMS group, including CAMs, ECM receptor interactions, and focal adhesion. Notably, CAMs play a vital role in cell-cell interactions, immune response modulation, and tumor cell migration. In GC, CAM-associated signaling is hyperactivated and strongly correlates with adverse clinical outcomes (62). Discoidin domain receptor 1 (DDR1) is a major ECM receptor. The upregulation of DDR1 in GC cells enhanced the metastatic ability of GC by promoting actin cytoskeleton reorganization (63). Focal adhesion drives tumor progression by regulating cell adhesion and migration, signal transduction, cytoskeletal reorganization and microenvironment interactions (64, 65). These pathways may reveal the potential mechanisms involved in the differences between high and low LYMS groups.

Tumor-infiltrating immune cells within the tumor microenvironment critically modulate tumor angiogenesis and LYM. Our findings demonstrated elevated immune cell infiltration in the high LYMS group, including macrophages, neutrophils, B cells, CD8+ T cells and mast cells. Notably, macrophages serve as direct structural contributors to the walls of lymphatic endothelial cells and secrete VEGF-C, VEGF-D, and VEGF-A to trigger LYM initiation in inflamed or tumor tissues (66). Tumor-associated neutrophils infiltrate tumor sites, where they secrete elevated levels of VEGF-A and MMP9, thereby driving tumor LYM and lymph node metastasis (67). B cells produce lymphangiogenic factors such as VEGF-A and VEGF-C, through synergistic signaling via B cell activating factor and IL-4 (68). VEGF-A produced by effector CD8+ T cells enhances T cell infiltration, tumor vascularization, and tumor progression (69). Furthermore, VEGF-A regulates CD8+ T cells by enhancing the expression of PD-1 and other inhibitory checkpoints involved in CD8+ T cell exhaustion (70). Mast cells also synthesize pro-lymphangiogenic factors VEGF-C and VEGF-D and pro-angiogenic factors VEGF-A, VEGF-B (71).

The tumor immune microenvironment plays a crucial role in tumor immunotherapy. Using the TIDE algorithm, we evaluated the function of immune cells in tumor microenvironment and their association with immunotherapy response. Elevated TIDE score usually indicates stronger immune evasion mechanisms in the tumor and poorer immunotherapy efficacy (72), which is associated with reduced response rate to immunotherapies such as PD-1/PD-L1 inhibitors and CTLA-4 inhibitors. TMB is also used to assess the response to immunotherapy. Emerging evidence indicates that tumor with elevated TMB generates increased neoantigens, which enhances T cell recognition and correlates with better outcomes following ICIs (such as PD-1/PD-L1 and CTLA-4 inhibitors) (73). This is consistent with our results, as the high LYMS group exhibited higher TIDE scores, lower TMB scores and lower IPS scores. These data collectively suggested that malignancies with high-LYMS had a greater potential for immune evasion, and patients in the high LYMS group might experience poorer outcomes with ICIs, which could explain the poor overall survival observed in high-LYMS patients. Overall, LYMS is a valuable indicator for predicting the response to immunotherapy in GC patients.

The drug sensitivity analysis indicated that patients in the high LYMS group showed resistance to common antitumor drugs (such as afatinib, gefitinib, dabrafenib, and lapatinib), but exhibited heightened sensitivity to antitumor drugs like dasatinib, NU7441, JAK8517, JQ1, OTX015 and alpelisib. Dasatinib, a SRC family kinases inhibitor, has shown efficacy in GC due to the overexpression of SRC. Choi et al. revealed that dasatinib modulated cellular energy homeostasis in GC and specifically targeted p90RSK (74). Additionally, dasatinib significantly enhances the cytotoxic effects of cisplatin by PI3K/AKT pathway (75) and oxaliplatin by suppressing Src activity triggered by oxaliplatin (76). NU7441, a DNA-dependent protein kinase inhibitor, can hinder the repair of DNA. Geng et al. found that NU7441 enhanced the susceptibility of radioresistant GC cells to radiotherapy by activating the caspase3/γH2AX signaling pathway (77). JQ1 and OTX015 are both small molecule inhibitors of the bromodomain and extraterminal. JQ1 suppresses the malignant progression of GC through reducing chromatin accessibility and inhibiting the RUNX2/NID1 signaling pathway (78). Alpelisib inhibites the proliferation of certain gastric cancer cells by suppressing PI3Kα (79). Furthermore, the combination of alpelisib and paclitaxel exhibits a synergistic anti-proliferative effect (80). Overall, drug sensitivity analysis offers guidance for clinical therapies, especially regarding antitumor drugs like dasatinib and alpelisib, which demonstrates enhanced therapeutic efficacy in GC patients with high levels of LYMS.

This study demonstrated that LYMS possessed promising predictive value for OS and provided guidance for clinical strategies in GC patients. Nonetheless, it is important to acknowledge the inherent limitations associated with retrospective studies, including selection bias and confounding bias. Therefore, it is important to carry out additional multicenter randomized controlled trials to validate these results. Therefore, conducting more multicenter randomized controlled trials and further validating these results in different patient cohorts and prospective studies is crucial. Additionally, the correlation between lymphangiogenesis and tumor metastasis is not a linear phenomenon, and tumor metastasis is also associated with various intrinsic factors of the tumor (such as EMT) and environmental factors. Our LYMS integrates gene expression from tissue transcriptomes. These transcriptomes contain a mixture of tumor cells, stromal cells, and immune infiltrating cells from both tumor and surrounding areas, making it spatially unable to distinguish between intratumoral and peritumoral lymphangiogenesis. Future studies integrating spatial transcriptomics may further elucidate the spatial specificity of LYMS. Future studies integrating spatial transcriptomics may further elucidate the spatial specificity of LYMS. In subsequent steps, we will further explore the interaction mechanisms between lymphangiogenesis and the tumor immune microenvironment, investigate the LYMS gene in particular cell lines and patient-derived xenograft models, and mechanistically clarify the association of LYMS with drug sensitivity. Overall, this research provides novel insights into the impact of LYM in the onset and development of GC, highlighting the need for further foundational studies to deepen our understanding of GC.

## 5 Conclusion

This article developed a LYMS model consisting of six genes, which showed good efficacy in forecasting the outcomes for GC patients. Additionally, we demonstrated the correlation of LYMS with the immune microenvironment and the immune therapy response in GC patients. Overall, LYMS operates as a forecast of risk for GC patients and can be utilized as a valuable tool in guiding immunotherapy decisions.

## Data Availability

The original contributions presented in the study are included in the article/**Supplementary Material**. Further inquiries can be directed to the corresponding author.
