# Supplementary material for: Development and verification of lymphangiogenesis score for prediction of prognosis and immune landscape in gastric cancer
Source: Front Immunol. 2025 Nov 4;16:1595592. doi: 10.3389/fimmu.2025.1595592 (PMC12623391; doi:10.3389/fimmu.2025.1595592)
Supplement: Supplementary file 1 [file Table1.docx]

Supplementary Material

**Supplementary table 1** The list of lymphangiogenesis -related genes

| VEGFC | FGFR3 | EIF4E | ITGAX | MUC1 |
| --- | --- | --- | --- | --- |
| FLT4 | TNFRSF1A | NKX2-1 | NOTCH4 | NRG1 |
| VEGFD | MYCN | TRPV1 | SOCS1 | PTK2 |
| VEGFA | EZR | SLIT2 | ILK | STAT2 |
| PDPN | IGFBP7 | TWIST1 | LGALS1 | TGFB3 |
| LYVE1 | CCL5 | CSF2 | RHOC | TYR |
| KDR | CD9 | PANX1 | CXCR5 | DLL4 |
| PROX1 | IGFBP1 | ROBO1 | LIMA1 | ETS1 |
| ANGPT2 | PDGFC | WNT5B | PIM3 | F8 |
| CALCRL | MACC1 | JAM3 | PTP4A3 | FBN1 |
| PTGS2 | MIR146A | BACH1 | BMP10 | FOLH1 |
| SOX18 | MIR155 | LMO2 | HMBOX1 | BECN1 |
| CALCRL-AS1 | MIR519E | HOXD8 | SRY | FZD6 |
| CCBE1 | MIR20B | FOXK1 | BCAR4 | GRB2 |
| LINC02577 | ACVRL1 | RASSF8 | MIR1468 | RUNX2 |
| VASH1 | NOS3 | JPT1 | MIR4466 | BMP6 |
| FLT1 | ITGA5 | HSP90AA2P | MIR4492 | BSG |
| FOXC2 | SEMA3A | MFSD4A-AS1 | BCL6 | CD274 |
| NRP2 | NTRK2 | SERPINE1 | AGGF1 | HAVCR2 |
| FGF2 | ITGA6 | PSIP1 | MCPH1 | TIMP1 |
| PGF | CPT1A | MIR494 | THBS1 | APLNR |
| PTPN14 | LEP | MIR655 | BRAF | CMA1 |
| TGFB1 | ADIPOQ | MMP14 | CDK5 | COX5A |
| CCR7 | DDR1 | ANXA2 | AR | CXCL10 |
| HIF1A | ADAMTS1 | EFNB2 | LDHA | CXCR3 |
| HMGB1 | TRAF2 | CYP26B1 | TNFSF11 | SKP2 |
| LINC02605 | ETV4 | SDC1 | ADAM9 | AGTR2 |
| POSTN | NOX4 | OLR1 | COL3A1 | INHA |
| CXCR4 | ID1 | NTN4 | PKD2 | LCP1 |
| CCL21 | SULF2 | GPR182 | PKD1 | SKIL |
| PDGFB | RIOX2 | MIA | PLXNA1 | SOD3 |
| MIR381 | ALKBH5 | MIR206 | ATG7 | TIMP3 |
| LINC00857 | MIR130A | NR3C1 | FGF4 | ADAMTSL1 |
| STAT3 | MIR199A1 | HOTTIP | FOXP2 | FABP5 |
| SHH | MIR199B | SNORD15A | ADAMTS2 | FRS2 |
| ANGPT1 | CTNNB1 | MIR648 | PLXNB1 | IL17F |
| BDNF-AS | GSK3B | MIR4754 | CCM2 | PTGES |
| NRP1 | JUN | MIR3178 | NDP | SOX17 |
| ERBB2 | RAC1 | MIR4491 | NPTX1 | CSF3 |
| LINC00958 | TBK1 | MIR4485 | PDCD10 | CXCL11 |
| NR2F2 | GLI1 | ENSG00000276609 | PELI1 | CXCL13 |
| MMP9 | FOXP3 | ENSG00000277469 | PPFIBP1 | MLANA |
| CXCL12 | IRS1 | ENSG00000277553 | LTB | MX1 |
| TEK | TGFBI | ENSG00000278708 | STYK1 | SPINT1 |
| ITGB1 | NECTIN4 | NCOR1 | ADAMTS14 | SRPX2 |
| VEGFB | IL13 | EFNA1 | GLMN | TIMP2 |
| TMX2-CTNND1 | TEAD4 | MIR373 | TSLP | CXCL9 |
| HPSE | VANGL1 | RUNX1-IT1 | NOVA2 | IAPP |
| MIR466 | CCL19 | MIR128-2 | IFITM2 | LGALS9 |
| HGF | CCN4 | PCNA | SVEP1 | PF4 |
| MET | MIR221 | THRB | SOX7 | PLXNC1 |
| CEACAM1 | MIR195 | FXYD5 | DEFB103B | SCARA5 |
| NFKB1 | RAMP2-AS1 | TBX1 | MIR126 | SENP2 |
| NOS2 | FAM225A | XIST | MIR185 | CLEC1B |
| S1PR1 | MIR300 | ENSG00000277577 | LOC117038771 | CCL8 |
| KRAS | ENSG00000273650 | MAPK1 | LOC123987613 | DUSP19 |
| PECAM1 | CALR | MYC | LOC126860291 | FGD5 |
| MIR507 | SRC | NGF | CDH5 | MIA3 |
| SMAD4 | IL1RN | EPHA2 | PTGS1 | APLN |
| IL17A | SPARC | BMPR2 | COL18A1 | CXCL14 |
| ITGA4 | BIRC3 | PPP3CB | ID2 | EMILIN2 |
| TNF | IL1A | FGFR2 | PCSK5 | NOX5 |
| FOXC1 | S1PR3 | SEMA7A | CBL | PTBP3 |
| IL7R | TBL1XR1 | SEMA3F | TNC | FOXF2 |
| SIX1 | NME1 | F2RL2 | ENG | KLHL6 |
| MIR27A | MAFB | CXCL5 | LPAR1 | CD24 |
| SMARCA4 | NR2F1 | SNORD118 | THBS2 | ADM2 |
| H19 | ARF6 | BCYRN1 | PCSK7 | GPR151 |
| IL6 | PAPSS1 | DUXAP9 | ACKR3 | MMRN2 |
| CLEC14A | MTA1 | LOC126805720 | LPAR3 | SFTA3 |
| MIR27B | TNFRSF6B | PIK3CA | RNF180 | DLEU1 |
| CERNA3 | SERPINE2 | INHBA | TRA-TGC7-1 | MEG3 |
| TIE1 | TNFAIP6 | RACK1 | TRA-TGC5-1 | RMRP |
| MAPK14 | CEBPD | DAB2IP | ENSG00000232995 | CYTOR |
| PDGFA | ANGPTL2 | LINC01672 | PLAU | HOXA11-AS |
| MCAM | PF4V1 | SEMA4D | HNF4A | JPX |
| MIR1236 | GAS5 | MKI67 | FURIN | MIR146B |
| TYMP | TRP-AGG2-5 | TNFSF15 | RB1 | MIR30B |
| EDN1 | TRP-AGG2-6 | PLG | CDH11 | MIR93 |
| HNF1A-AS1 | TRP-AGG2-1 | KRIT1 | PROM1 | SNHG7 |
| MIR9-1 | TRP-AGG2-2 | JAK1 | TRAF6 | MIR19A |
| IL24 | TRP-AGG2-3 | BCL2 | ARNT | MIR337 |
| SMAD5-AS1 | TRP-AGG2-4 | JAK3 | CNTN1 | SNHG1 |
| TIAM1 | TRP-AGG2-7 | FN1 | ISG15 | SNHG6 |
| ECM1 | TRP-AGG2-8 | IGF2 | SP1 | HAS2-AS1 |
| LIMS1 | ACVR2B | MAPK3 | SPHK1 | LEF1-AS1 |
| NES | MTOR | ITGA2 | TGFA | LINC00092 |
| CDKN2B-AS1 | RELA | ITGA1 | CRK | MIR215 |
| ADM | NFKBIA | PLPP3 | ALCAM | MIR31HG |
| ITGA9 | CDKN1A | HOXD10 | GDF15 | MIR424 |
| GATA2 | CAV1 | SEMA4C | GREM1 | SNHG17 |
| MMP2 | HDAC5 | MIR181D | PCSK6 | CASC15 |
| IGF1 | HNRNPA1 | RNU6-1 | RAMP2 | DLEU7-AS1 |
| NFATC1 | ACTN4 | RNU6-2 | SP3 | MIR144 |
| SCARNA5 | CSNK1E | MIR559 | NKX3-1 | MIR302B |
| AREG | LAMC2 | RNU6-7 | RAMP3 | MIR7-3 |
| IL7 | BMX | RNU6-8 | LBX1 | PCAT1 |
| CXCL8 | PRMT5 | RNU6-9 | MUC20 | MIR153-2 |
| PDGFRB | LCN2 | RNU6-1-001 | SPRR1B | MIR7-2 |
| SERPINA4 | PFKFB3 | RNU6-1-002 | MIR7-3HG | CASC8 |
| RHOA | CD151 | RNU6-1-003 | MIR133B | CASC9 |
| NOTCH1 | F2RL1 | RNU6-1-004 | MIR532 | LINC01089 |
| LTBR | MYO5A | CXCR2 | LINC01186 | MIR7-1 |
| NCAM1 | NLRC4 | PIK3R1 | LOC100131635 | MIR942 |
| TGFBR1 | TSG101 | TSC2 | NPTN-IT1 | PCAT2 |
| AKT1 | KRT10 | CDKN1B | FLJ42393 | SOX9-AS1 |
| ELAVL1 | ZIC2 | EPCAM | MIR548K | CASC19 |
| EPAS1 | PABPC4 | GPI | MIR4306 | CCAT1 |
| RETN | PRPF6 | GATA6 | ENSG00000275307 | MIR153-1 |
| MIR186 | RAB10 | GHR | ENSG00000276965 | MIR663B |
| YAP1 | SYCP3 | PLA2G2A | EGFR | PWAR1 |
| IL4 | KPNA4 | PTGER4 | IGF1R | RAB4B-EGLN2 |
| PTGER1 | DDX17 | ANG | EPHB4 | SNORD50A |
| EMSLR | RPL6 | GDF2 | PDGFRA | LINC01093 |
| LGALS8 | ESM1 | PTPRM | PIK3CD | MIR4437 |
| CDH1 | ELK3 | GH1 | TP53 | ROCR |
| BDNF | U2AF2 | PLA2G10 | HMOX1 | MIR493HG |
| CCN2 | TRIM3 | CCL26 | HSP90AA1 | SOD2-OT1 |
| IL1B | SERBP1 | STAU1 | NFE2L2 | CCL15-CCL14 |
| SIRT2 | AKIP1 | NTAN1 | PTEN | ENSG00000276919 |
| LOXL2 | MIR204 | FNDC1 | TERT | ENSG00000288605 |
| RSPO2 | RMST | SNHG16 | TGFB2 | ENSG00000273961 |
| MIR624 | MIR182 | CREBBP | HDAC1 | ENSG00000276496 |
| PGR-AS1 | MIR217 | MAP2K1 | MMP13 | ENSG00000277966 |
| MIR526B | MIR503 | RAF1 | CD36 | ENSG00000278020 |
| CDKN2A | MIR296 | AGTR1 | MAPK10 | ENSG00000278334 |
| EDNRB | MIR129-1 | TLR4 | MAPK8 | ENSG00000278592 |
| FASN | RPL13AP20 | ADAM17 | NOS1 | HAFML |
| SEMA3C | MIR10527 | SYK | RPS6KB1 | ENSG00000274430 |
| CD82 | FGFR1 | CCL2 | SIRT1 | ENSG00000276784 |
| ADAMTS3 | EGF | PIM1 | CCNE1 | LOC102723517 |
| EMILIN1 | APOA1 | RHO | CD44 | LOC108281116 |
