# Supplementary material for: Development and verification of lymphangiogenesis score for prediction of prognosis and immune landscape in gastric cancer
Source: Front Immunol. 2025 Nov 4;16:1595592. doi: 10.3389/fimmu.2025.1595592 (PMC12623391; doi:10.3389/fimmu.2025.1595592)
Supplement: Supplementary file 2 [file Table2.docx]

Supplementary Material

**Supplementary table 2** The list of primer for quantitative real time PCR

| **name** |  | **Primer5-3** |
| --- | --- | --- |
| ADAMTS1(H) | F | CTTGTGGCAGACCAGTCGAT |
|  | R | TTCACCACCACCAGGCTAAC |
| CAV1(H) | F | AGATTGACTTTGAAGATGTGATT |
|  | R | ACAGTGAAGGTGGTGAAG |
| NOX4(H) | F | AGGAGAACCAGGAGATTGTTG |
|  | R | GGGATGACTTATGACCGAAAT |
| NPTX1(H) | F | ACCGAGGAGAGGGTCAAGAT |
|  | R | GTGGGAATGTGAGCTGGAAC |
| SVEP1(H) | F | GTTGCATTGAGGAGTTAGCATT |
|  | R | CTGTTAGCAAGACAGGATGATT |
| SPARC(H) | F | TTCCTGCCACTTCTTTGCCA |
|  | R | TTTGCAAGGCCCGATGTAGT |
| β-actin(H) | F | CTTCGCGGGCGACGAT |
|  | R | CACATAGGAATCCTTCTGACCCAT |
