# Supplementary material for: Development and verification of lymphangiogenesis score for prediction of prognosis and immune landscape in gastric cancer
Source: Front Immunol. 2025 Nov 4;16:1595592. doi: 10.3389/fimmu.2025.1595592 (PMC12623391; doi:10.3389/fimmu.2025.1595592)
Supplement: Supplementary file 3 [file Table3.docx]

Supplementary Material

**Supplementary table 3** The list of differentially expressed genes.

| **ID** | **logFC** | **AveExpr** | **t** | **P.Value** | **adj.P.Val** | **B** |
| --- | --- | --- | --- | --- | --- | --- |
| SCARA5 | -4.38136 | 5.699373 | -18.9063 | 4.05E-59 | 1.93E-56 | 123.8313 |
| ADIPOQ | -4.11912 | 0.436356 | -8.61308 | 1.21E-16 | 1.74E-15 | 27.10325 |
| VEGFD | -3.94186 | 3.143229 | -16.8774 | 7.03E-50 | 1.12E-47 | 102.6435 |
| CMA1 | -3.54046 | 1.050863 | -10.062 | 1.28E-21 | 3.21E-20 | 38.34789 |
| RSPO2 | -3.4236 | 3.711635 | -11.2418 | 5.15E-26 | 2.46E-24 | 48.26687 |
| NPTX1 | -3.19584 | 4.612767 | -8.73602 | 4.78E-17 | 7.60E-16 | 27.85604 |
| NCAM1 | -3.15545 | 6.452279 | -14.2428 | 2.88E-38 | 2.74E-36 | 76.09214 |
| LYVE1 | -3.01159 | 6.11627 | -17.6927 | 1.43E-53 | 3.41E-51 | 111.1342 |
| NOS1 | -3.00257 | 3.118108 | -8.82048 | 2.52E-17 | 4.14E-16 | 28.59235 |
| AGTR1 | -2.92024 | 3.465048 | -7.71364 | 7.92E-14 | 7.13E-13 | 20.6733 |
| APOA1 | -2.86017 | 4.608886 | -5.57597 | 4.25E-08 | 1.93E-07 | 7.691334 |
| CNTN1 | -2.79007 | 5.58373 | -8.61967 | 1.15E-16 | 1.71E-15 | 26.93132 |
| CCL21 | -2.54309 | 8.343429 | -7.94943 | 1.52E-14 | 1.51E-13 | 21.85583 |
| GHR | -2.48738 | 5.842852 | -13.7499 | 3.61E-36 | 2.87E-34 | 71.35898 |
| TSLP | -2.41226 | 3.371239 | -10.1484 | 6.24E-22 | 1.75E-20 | 39.03146 |
| ADAMTSL1 | -2.32088 | 6.745223 | -16.4653 | 5.00E-48 | 5.96E-46 | 98.44232 |
| FOXP2 | -2.2273 | 6.525985 | -8.08019 | 5.99E-15 | 6.65E-14 | 22.98114 |
| PTGS1 | -2.07653 | 8.895973 | -13.3807 | 1.29E-34 | 8.77E-33 | 67.64526 |
| CD36 | -2.06033 | 7.86723 | -11.1455 | 1.20E-25 | 5.23E-24 | 47.19216 |
| LPAR3 | -2.02645 | 2.854526 | -6.11995 | 2.04E-09 | 1.06E-08 | 10.84179 |
| AR | -1.90575 | 5.608435 | -8.52389 | 2.35E-16 | 3.29E-15 | 26.29438 |
| CXCR2 | -1.89768 | 4.872507 | -6.61445 | 1.06E-10 | 6.48E-10 | 13.59054 |
| CXCL14 | -1.87973 | 10.04841 | -5.30208 | 1.80E-07 | 7.27E-07 | 5.864312 |
| FOXF2 | -1.80329 | 7.919694 | -9.57671 | 6.75E-20 | 1.40E-18 | 34.1015 |
| AGTR2 | -1.79941 | -0.89289 | -5.19613 | 3.09E-07 | 1.23E-06 | 6.063482 |
| ADAMTS1 | -1.79437 | 9.707724 | -9.98458 | 2.43E-21 | 5.79E-20 | 37.28053 |
| NRG1 | -1.76447 | 6.078494 | -6.13647 | 1.85E-09 | 9.70E-09 | 10.67735 |
| MAPK10 | -1.73969 | 6.616701 | -10.1738 | 5.05E-22 | 1.51E-20 | 39.08066 |
| CXCL12 | -1.70859 | 9.281418 | -8.38346 | 6.63E-16 | 8.79E-15 | 24.92612 |
| BMP6 | -1.69023 | 5.924363 | -9.95073 | 3.21E-21 | 7.29E-20 | 37.30362 |
| FGF2 | -1.67762 | 6.4003 | -8.34781 | 8.62E-16 | 1.06E-14 | 24.95567 |
| BMX | -1.61348 | 4.112219 | -7.4994 | 3.44E-13 | 2.98E-12 | 19.25948 |
| SLIT2 | -1.6031 | 7.346878 | -6.14284 | 1.78E-09 | 9.45E-09 | 10.58054 |
| CCBE1 | -1.59997 | 4.174662 | -6.90678 | 1.70E-11 | 1.19E-10 | 15.44832 |
| LPAR1 | -1.57392 | 7.952985 | -10.3492 | 1.16E-22 | 4.25E-21 | 40.41042 |
| ID1 | -1.56146 | 10.47055 | -7.05455 | 6.57E-12 | 4.89E-11 | 15.83062 |
| RNF180 | -1.4074 | 5.749961 | -8.3468 | 8.68E-16 | 1.06E-14 | 25.02727 |
| CDKN1A | -1.39348 | 10.9934 | -9.46137 | 1.70E-19 | 3.24E-18 | 33.04934 |
| CAV1 | -1.37722 | 10.33304 | -6.89493 | 1.83E-11 | 1.26E-10 | 14.83465 |
| SOX17 | -1.3692 | 6.060514 | -9.17694 | 1.61E-18 | 2.84E-17 | 31.19886 |
| PIM1 | -1.34604 | 9.833299 | -10.2301 | 3.15E-22 | 1.00E-20 | 39.31155 |
| WNT5B | -1.33712 | 6.777746 | -6.38158 | 4.36E-10 | 2.36E-09 | 12.05545 |
| SOD3 | -1.31322 | 10.33666 | -5.8679 | 8.56E-09 | 4.17E-08 | 8.826263 |
| JAM3 | -1.31095 | 7.748227 | -7.52908 | 2.81E-13 | 2.48E-12 | 19.13032 |
| CEBPD | -1.27713 | 11.19388 | -8.35043 | 8.45E-16 | 1.06E-14 | 24.63691 |
| JUN | -1.26298 | 12.02734 | -7.96637 | 1.35E-14 | 1.37E-13 | 21.90011 |
| CXCR5 | -1.24563 | 1.230311 | -3.28535 | 0.001098 | 0.00258 | -1.69087 |
| NOX5 | -1.22256 | 2.043357 | -4.23423 | 2.78E-05 | 8.34E-05 | 1.735364 |
| ANGPT1 | -1.20361 | 6.380748 | -7.37086 | 8.18E-13 | 6.61E-12 | 18.26062 |
| THRB | -1.19998 | 8.077979 | -4.74381 | 2.82E-06 | 1.00E-05 | 3.3961 |
| TBX1 | -1.19361 | 4.556593 | -4.52719 | 7.66E-06 | 2.50E-05 | 2.855721 |
| BCL2 | -1.17809 | 7.951906 | -8.05499 | 7.18E-15 | 7.76E-14 | 22.7213 |
| ITGA9 | -1.17065 | 8.466423 | -6.29087 | 7.48E-10 | 4.01E-09 | 11.33549 |
| MAPK3 | -1.13759 | 11.2812 | -9.07402 | 3.58E-18 | 6.11E-17 | 30.0324 |
| NR2F1 | -1.12874 | 8.566867 | -5.61812 | 3.39E-08 | 1.55E-07 | 7.614369 |
| NTRK2 | -1.11866 | 6.575745 | -3.47139 | 0.000568 | 0.001433 | -1.39984 |
| PTGER1 | -1.10358 | 4.950446 | -4.3288 | 1.85E-05 | 5.69E-05 | 1.983941 |
| CCL19 | -1.09896 | 6.053445 | -2.82387 | 0.004955 | 0.010101 | -3.35809 |
| LEP | -1.08212 | 1.655645 | -3.35747 | 0.000853 | 0.002045 | -1.46217 |
| SRY | -1.06525 | -1.8045 | -4.53407 | 7.43E-06 | 2.44E-05 | 3.046658 |
| SVEP1 | -1.05879 | 8.207071 | -4.85142 | 1.69E-06 | 6.21E-06 | 3.877132 |
| IL17F | -1.0537 | -0.00933 | -2.9612 | 0.003226 | 0.006809 | -2.64898 |
| PLPP3 | -1.0509 | 9.673113 | -10.6078 | 1.29E-23 | 5.11E-22 | 42.49911 |
| TIMP3 | -1.04975 | 12.05596 | -4.97289 | 9.40E-07 | 3.53E-06 | 4.24385 |
| ACKR3 | -1.04713 | 8.431577 | -7.24979 | 1.83E-12 | 1.43E-11 | 17.23827 |
| TIAM1 | -1.04105 | 7.074964 | -5.41411 | 1.00E-07 | 4.31E-07 | 6.761097 |
| NTN4 | -1.04074 | 8.927365 | -6.82863 | 2.78E-11 | 1.84E-10 | 14.51576 |
| IL1RN | -1.03652 | 9.59208 | -3.18491 | 0.001548 | 0.003468 | -2.70698 |
| DUSP19 | -1.02451 | 6.386568 | -6.47118 | 2.54E-10 | 1.41E-09 | 12.65953 |
| NR3C1 | -1.02179 | 9.68504 | -7.83189 | 3.48E-14 | 3.39E-13 | 21.03146 |
| PLXNA1 | 1.006869 | 10.89978 | 8.194681 | 2.63E-15 | 2.99E-14 | 23.61304 |
| SKP2 | 1.031743 | 9.285292 | 4.817859 | 1.99E-06 | 7.23E-06 | 3.814533 |
| PF4V1 | 1.049069 | 0.652764 | 2.975423 | 0.003083 | 0.006536 | -2.58825 |
| NME1 | 1.057336 | 9.086617 | 4.365591 | 1.57E-05 | 4.94E-05 | 1.855957 |
| SHH | 1.059911 | 7.024848 | 2.472088 | 0.013801 | 0.025715 | -4.17682 |
| IL1A | 1.060404 | 4.093601 | 2.879894 | 0.004168 | 0.008643 | -2.92082 |
| PCNA | 1.089719 | 11.21151 | 6.402224 | 3.85E-10 | 2.11E-09 | 11.90839 |
| FN1 | 1.101171 | 14.13425 | 4.438601 | 1.14E-05 | 3.63E-05 | 1.846707 |
| HAVCR2 | 1.133567 | 7.586275 | 5.325709 | 1.59E-07 | 6.54E-07 | 6.470522 |
| ITGA2 | 1.134228 | 10.47864 | 5.456745 | 8.02E-08 | 3.57E-07 | 6.77764 |
| PDGFRB | 1.177138 | 11.4954 | 7.017869 | 8.32E-12 | 6.11E-11 | 15.65542 |
| CDH11 | 1.181942 | 10.19067 | 5.775545 | 1.43E-08 | 6.90E-08 | 8.485829 |
| SPARC | 1.184275 | 13.71864 | 7.461342 | 4.45E-13 | 3.79E-12 | 18.45993 |
| LOXL2 | 1.23126 | 9.987236 | 7.298581 | 1.32E-12 | 1.05E-11 | 17.59256 |
| HOXD10 | 1.234354 | 1.5045 | 2.372597 | 0.018083 | 0.032514 | -4.17507 |
| TEAD4 | 1.284332 | 8.712218 | 6.727844 | 5.24E-11 | 3.33E-10 | 14.15852 |
| TIMP1 | 1.321684 | 13.08439 | 7.766226 | 5.50E-14 | 5.05E-13 | 20.52881 |
| FOXP3 | 1.467526 | 6.556846 | 6.488391 | 2.29E-10 | 1.28E-09 | 12.92164 |
| ANGPT2 | 1.506317 | 7.844322 | 6.791534 | 3.52E-11 | 2.30E-10 | 14.65911 |
| CCNE1 | 1.561178 | 7.551149 | 4.083451 | 5.25E-05 | 0.000152 | 0.966136 |
| MET | 1.575629 | 10.9383 | 7.098382 | 4.94E-12 | 3.74E-11 | 16.22981 |
| SERPINE1 | 1.606166 | 10.21297 | 5.708166 | 2.07E-08 | 9.70E-08 | 8.1808 |
| PLAU | 1.606781 | 9.521924 | 6.649552 | 8.53E-11 | 5.35E-10 | 13.60917 |
| CDKN2A | 1.607243 | 7.269089 | 3.130199 | 0.00186 | 0.004071 | -2.33503 |
| ITGAX | 1.613055 | 8.571292 | 7.420514 | 5.86E-13 | 4.90E-12 | 18.6001 |
| COL3A1 | 1.613933 | 14.6932 | 8.052177 | 7.32E-15 | 7.76E-14 | 22.50214 |
| F2RL2 | 1.709209 | 8.008765 | 5.555918 | 4.73E-08 | 2.13E-07 | 7.64957 |
| SIX1 | 1.829087 | 5.015183 | 5.066559 | 5.92E-07 | 2.26E-06 | 5.394737 |
| MACC1 | 1.842397 | 10.06207 | 6.096838 | 2.33E-09 | 1.19E-08 | 10.32654 |
| MMP9 | 1.958251 | 8.752629 | 5.167283 | 3.58E-07 | 1.41E-06 | 5.630667 |
| CXCL10 | 1.965964 | 8.244883 | 4.296437 | 2.13E-05 | 6.51E-05 | 1.778998 |
| LAMC2 | 1.972006 | 11.26803 | 6.893318 | 1.85E-11 | 1.26E-10 | 14.93962 |
| CCN4 | 2.078986 | 7.804536 | 7.812588 | 3.98E-14 | 3.80E-13 | 21.33542 |
| CXCL5 | 2.090948 | 8.220708 | 2.501866 | 0.012708 | 0.023959 | -4.13568 |
| CXCL11 | 2.117221 | 6.760752 | 4.665814 | 4.06E-06 | 1.38E-05 | 3.499558 |
| ADAMTS14 | 2.1393 | 7.354364 | 8.725345 | 5.18E-17 | 7.98E-16 | 27.88032 |
| THBS2 | 2.1463 | 9.826679 | 5.407748 | 1.04E-07 | 4.38E-07 | 6.73896 |
| TERT | 2.163881 | 4.139334 | 5.746165 | 1.68E-08 | 8.03E-08 | 8.837942 |
| IL24 | 2.166097 | 3.313391 | 5.423125 | 9.57E-08 | 4.15E-07 | 7.175896 |
| NOX4 | 2.172898 | 5.554557 | 9.808561 | 1.03E-20 | 2.23E-19 | 36.29813 |
| CXCL9 | 2.254889 | 9.630598 | 4.743776 | 2.82E-06 | 1.00E-05 | 3.571586 |
| APLN | 2.256778 | 6.635242 | 5.937792 | 5.78E-09 | 2.84E-08 | 9.824407 |
| EPCAM | 2.257044 | 12.95592 | 9.541396 | 8.96E-20 | 1.78E-18 | 33.71513 |
| ADAMTS2 | 2.328723 | 9.872321 | 8.2796 | 1.42E-15 | 1.69E-14 | 24.45133 |
| TNFSF11 | 2.513678 | 4.978813 | 9.37213 | 3.46E-19 | 6.34E-18 | 32.85172 |
| IGFBP1 | 2.527695 | 2.150557 | 4.903242 | 1.32E-06 | 4.87E-06 | 4.682343 |
| ETV4 | 2.53508 | 8.848163 | 6.013393 | 3.76E-09 | 1.89E-08 | 10.09053 |
| MKI67 | 2.54528 | 11.79492 | 11.35007 | 1.97E-26 | 1.04E-24 | 48.9821 |
| HNF4A | 2.565267 | 10.94063 | 6.978037 | 1.08E-11 | 7.77E-11 | 15.53736 |
| CXCL8 | 2.572812 | 9.348795 | 4.724922 | 3.08E-06 | 1.09E-05 | 3.572937 |
| CSF2 | 2.661926 | 2.085165 | 6.560187 | 1.48E-10 | 8.60E-10 | 13.45743 |
| OLR1 | 2.716223 | 6.562811 | 8.201614 | 2.50E-15 | 2.91E-14 | 24.1234 |
| MMP13 | 2.735482 | 3.241028 | 6.634911 | 9.34E-11 | 5.78E-10 | 13.89138 |
| ZIC2 | 2.910573 | 4.494394 | 4.540326 | 7.22E-06 | 2.39E-05 | 3.025567 |
| GDF15 | 2.921844 | 9.896322 | 7.40222 | 6.63E-13 | 5.45E-12 | 18.45634 |
| FNDC1 | 3.496143 | 9.166702 | 6.58351 | 1.28E-10 | 7.55E-10 | 13.4294 |
| INHBA | 3.669769 | 9.530081 | 10.23797 | 2.95E-22 | 1.00E-20 | 39.7349 |
| ESM1 | 4.266005 | 6.298917 | 12.0021 | 5.48E-29 | 3.27E-27 | 55.04791 |
