# Supplementary material for: Development and verification of lymphangiogenesis score for prediction of prognosis and immune landscape in gastric cancer
Source: Front Immunol. 2025 Nov 4;16:1595592. doi: 10.3389/fimmu.2025.1595592 (PMC12623391; doi:10.3389/fimmu.2025.1595592)
Supplement: Supplementary file 4 [file DataSheet1.docx]

Supplementary Material

#
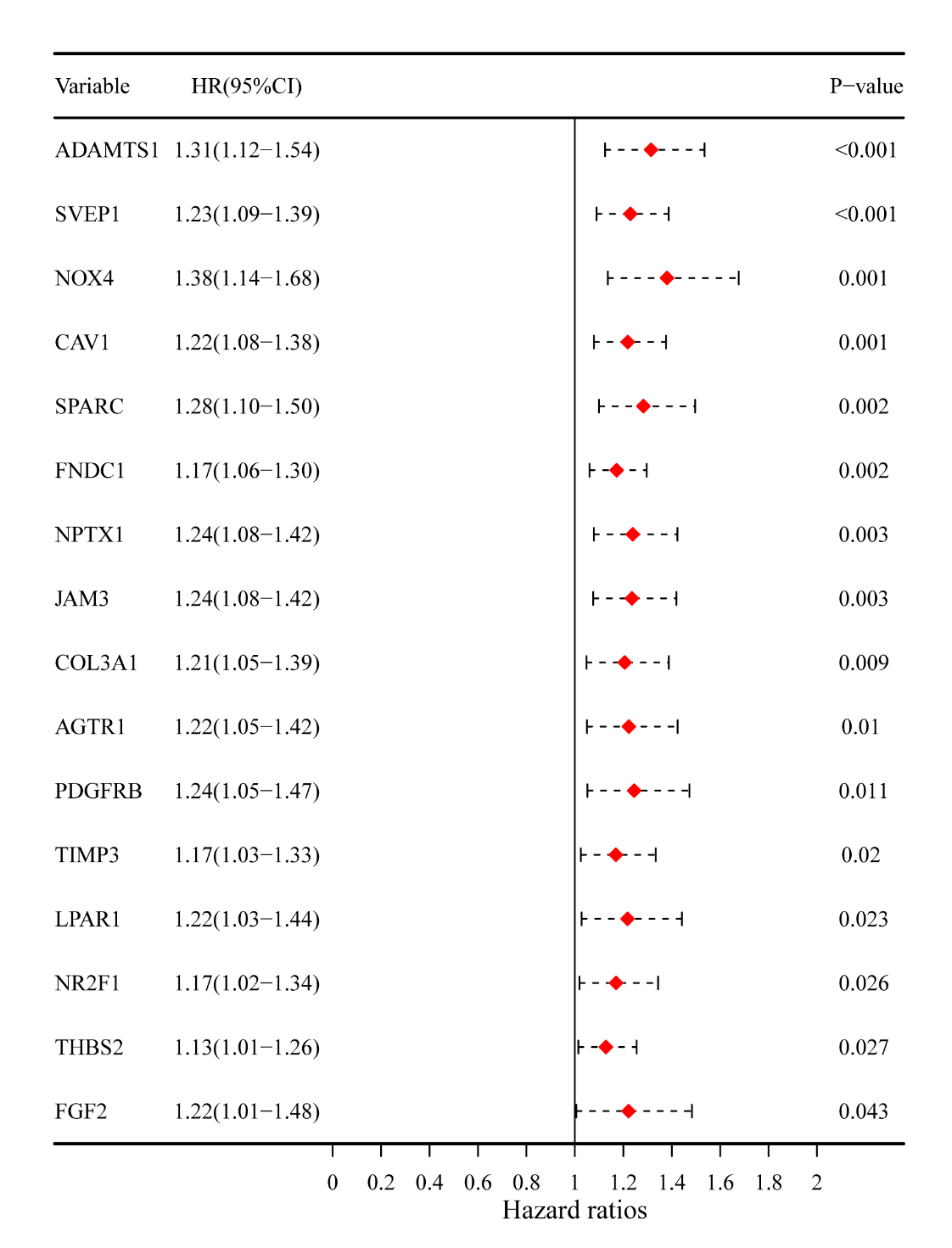
Supplementary Figures

**Supplementary Figure 1.** **Forest plot for intersection results of univariate cox regression analysis.**

**
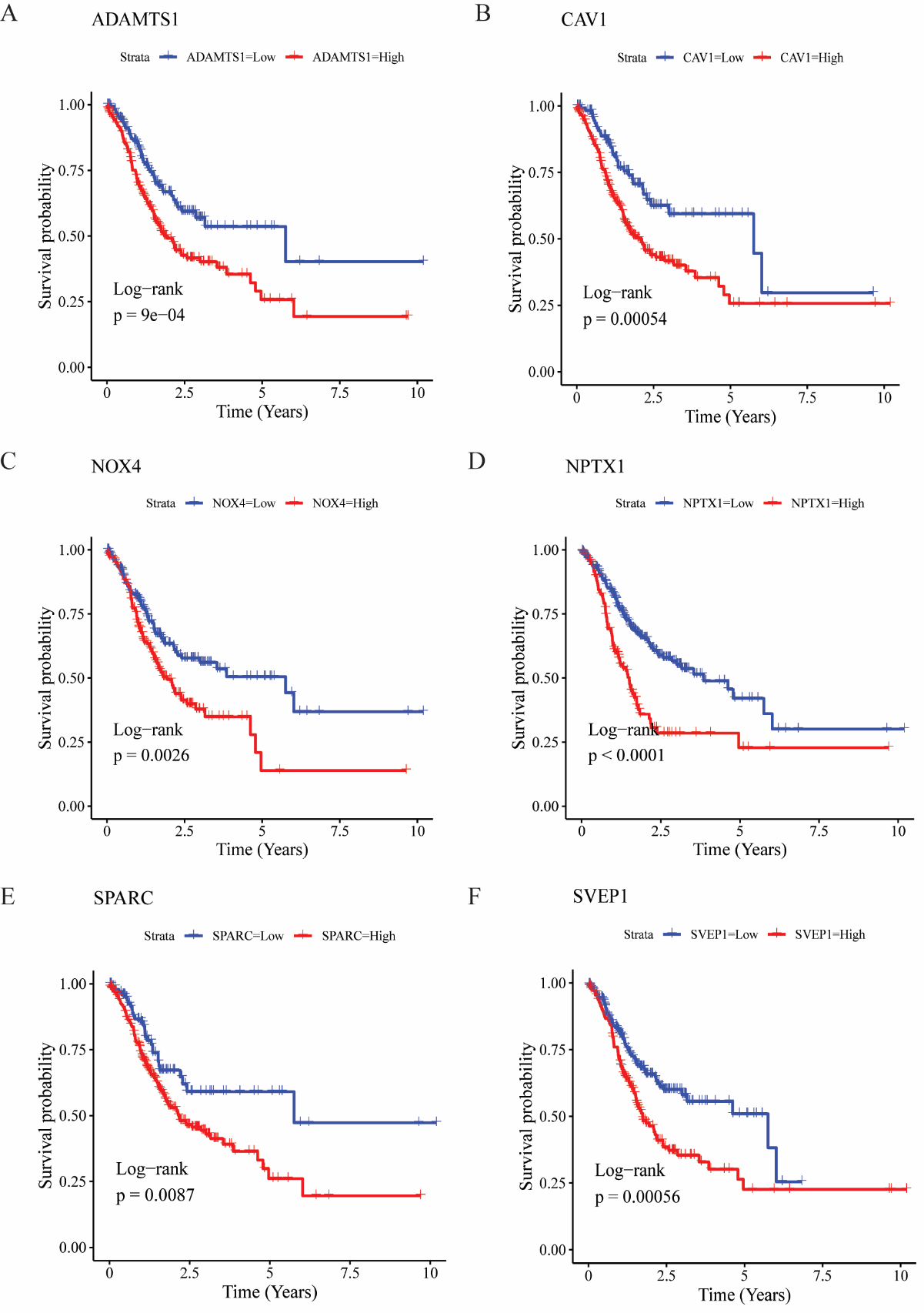
**

**Supplementary Figure 2.** **Kaplan-Meier analysis of each model genes.** (A) ADAMTS1, (B)CAV1, (C) NOX4, (D) NPTX1, (E) SPARC, (F)SVEP1.


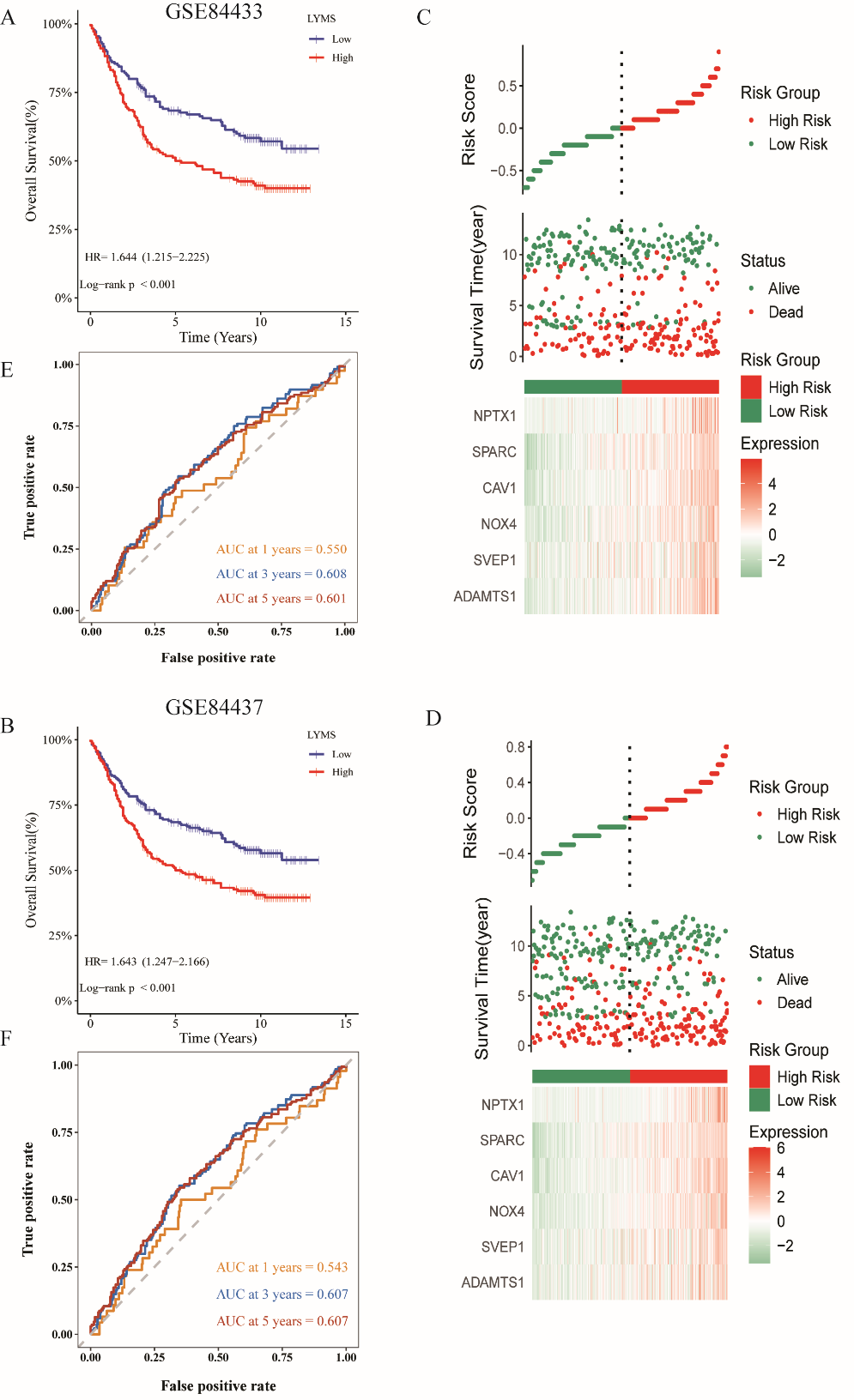


**Supplementary Figure 3.** **External validation** **of a prognostic gene signature for GC patients.** (A, B) The KM analysis of LYMS predicting the OS of patients in GSE84433 and GSE84437 cohorts. (C, D) Distribution of risk score, survival status and heatmap of LYMS including 6 genes in GSE84433 and GSE84437 cohorts. (E, F) The ROC curves evaluating the predictive accuracy of LYMS at 1-,3- and 5-years in GSE84433 and GSE84437 cohorts. LYMS, lymphangiogenesis score; KM, Kaplan-Meier; AUC, areas under the curve.


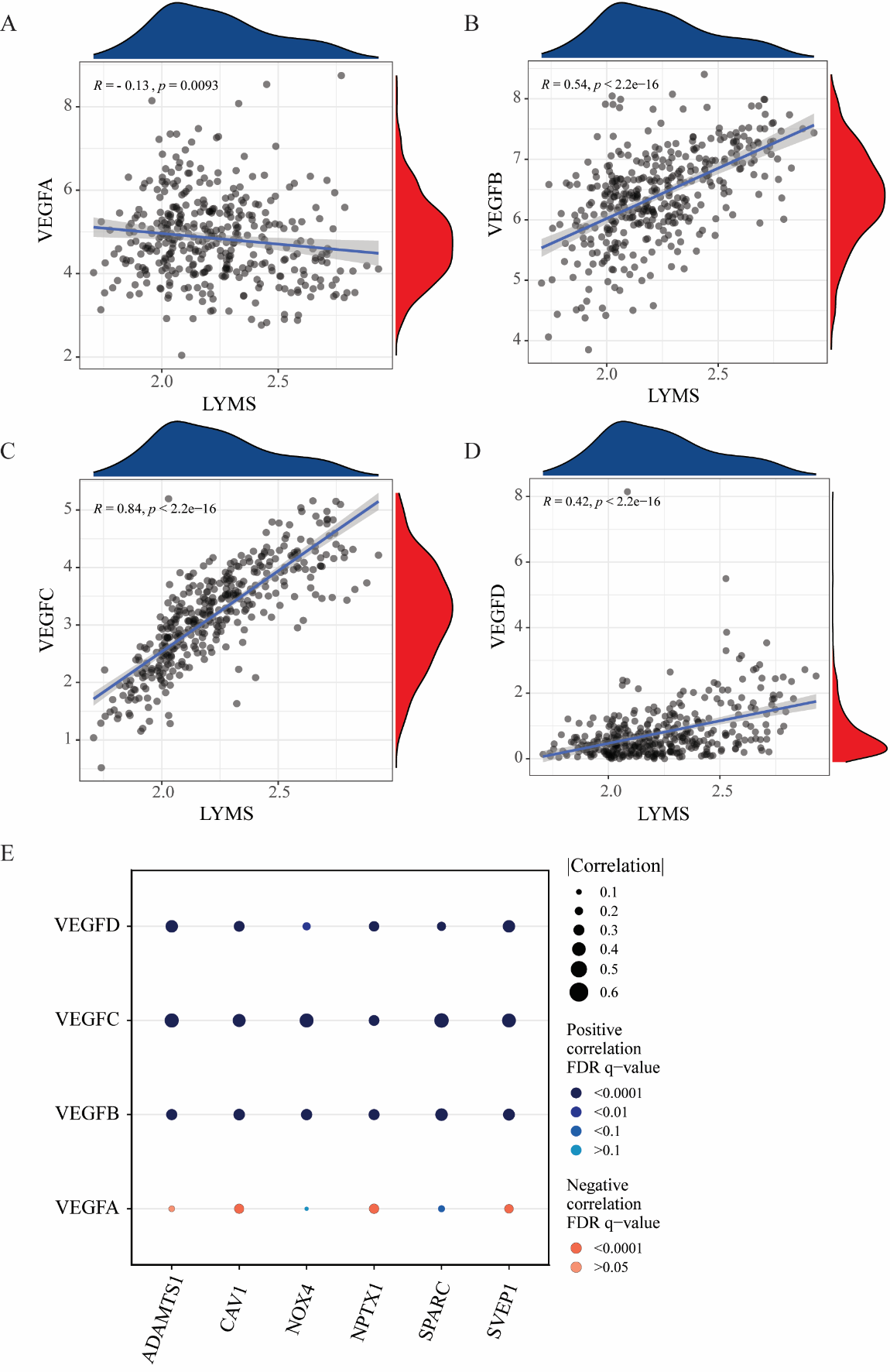


**Supplementary Figure 4. The correlation between the LYMS and various VEGFs.** (A) VEGFA, (B) VEGFB, (C) VEGFC, (D) VEGFD, (E) The correlation between each model genes and VEGFs. VEGF, vascular endothelial growth factor.


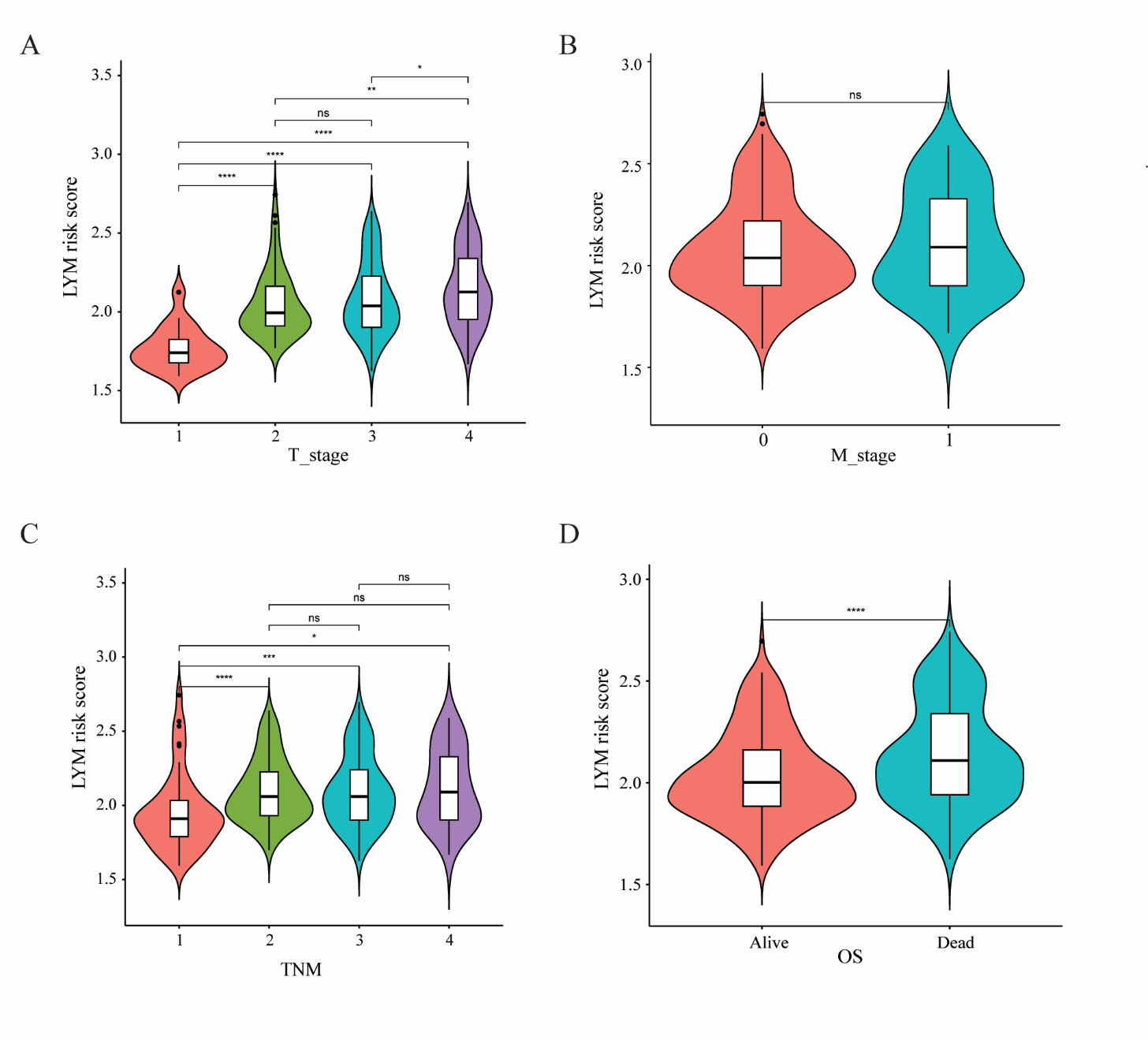


**Supplementary Figure 5. The correlation between the LYMS and clinical characteristics.** (A) T stage, (B) M stage, (C)TNM stage and (D) OS in GC patients.


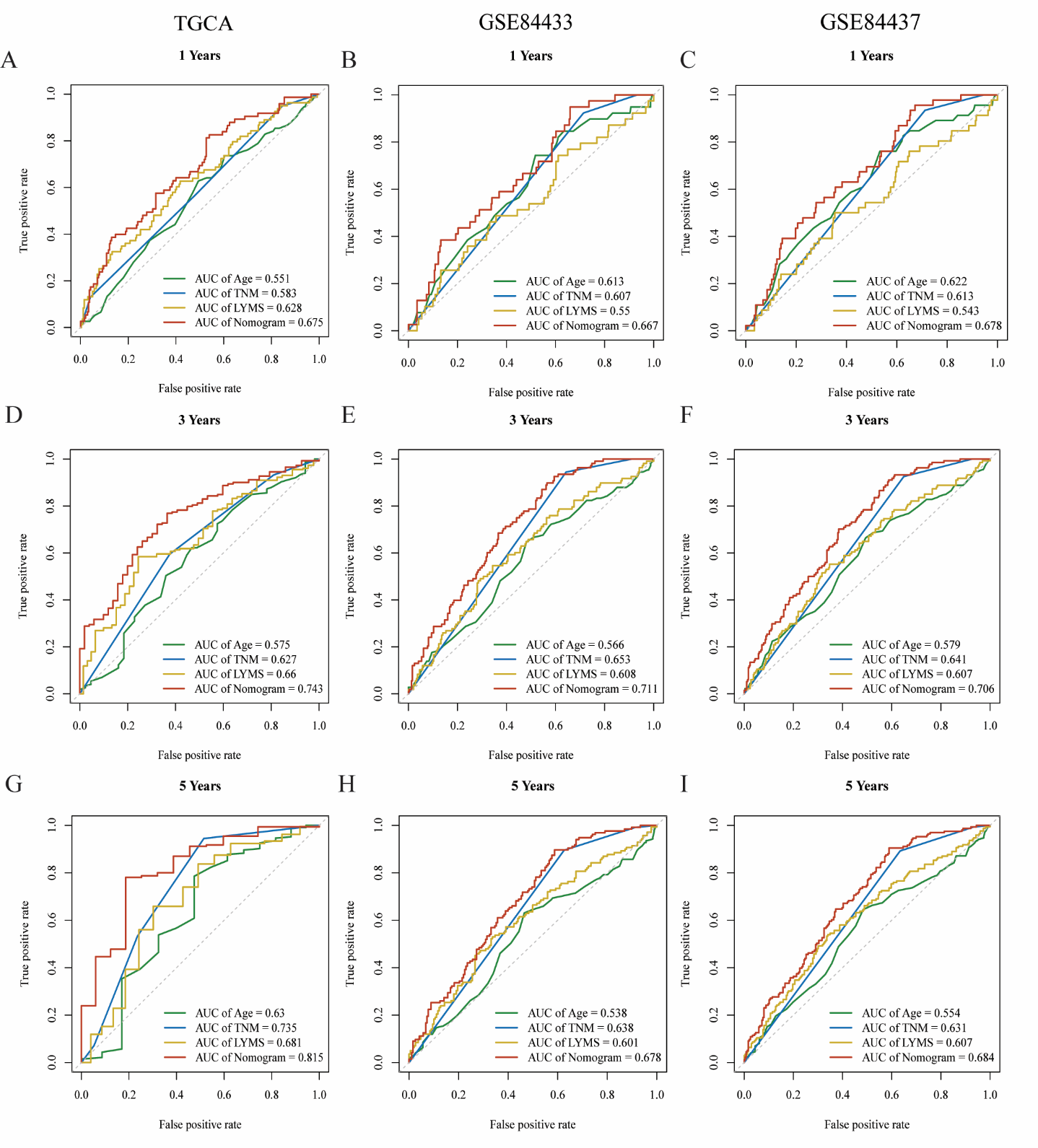


**Supplementary Figure 6. Time dependent ROC curves for different models in GC patients.** (A, B, C) Time dependent ROC curves at 1 year for different models in GC patients in TCGA, GSE84433 and GSE84437 cohorts. (D, E, F) Time dependent ROC curves at 3 years for different models in GC patients in TCGA, GSE84433 and GSE84437 cohorts. (G, H, I) Time dependent ROC curves at 5 years for different models in GC patients in TCGA, GSE84433 and GSE84437 cohorts. AUC, areas under the curve.


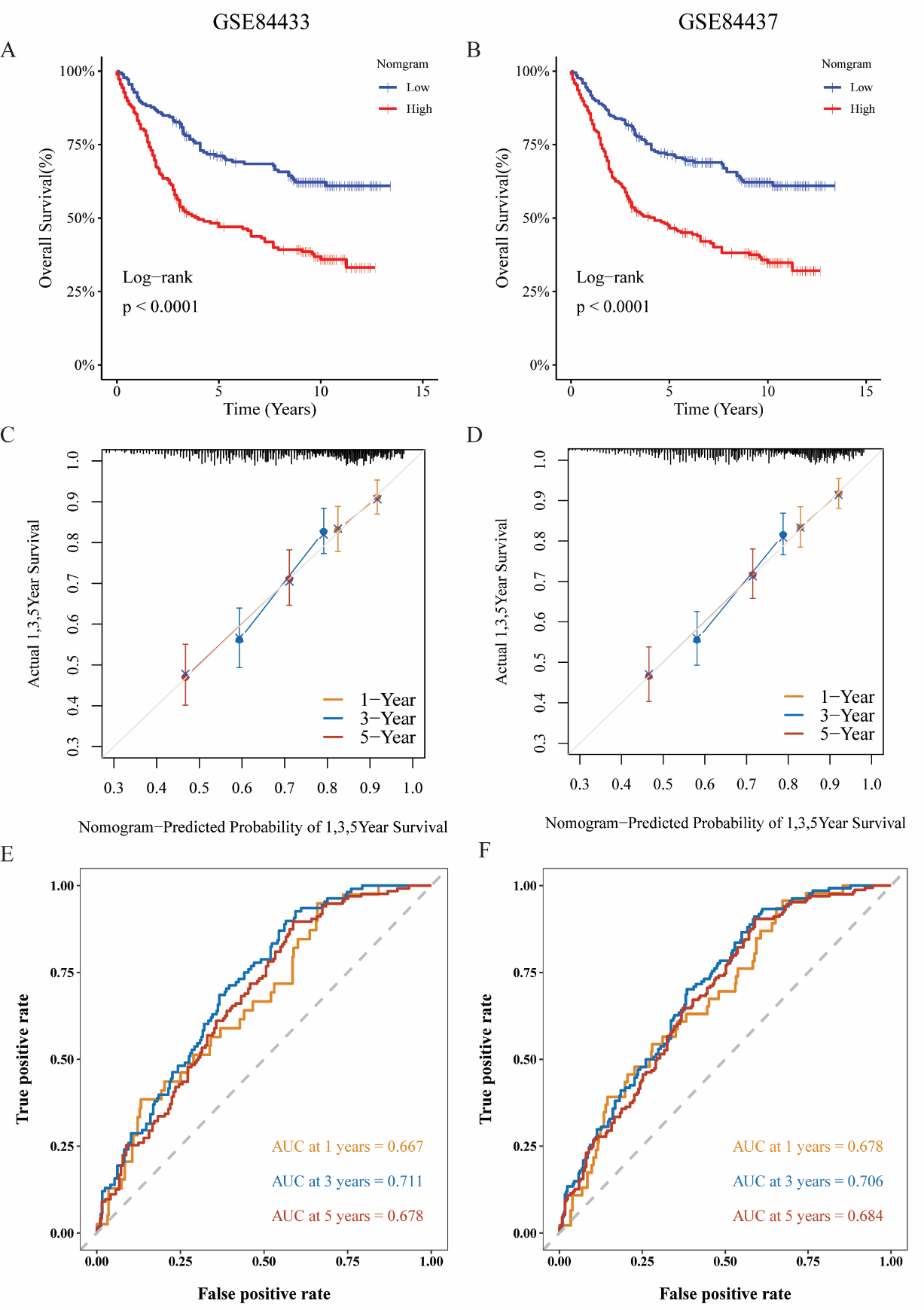


**Supplementary Figure 7.** **External validation of the nomogram survival model.** (A, B) Kaplan-Meier analyses for the two groups based on the nomogram in GSE84433 and GSE84437 cohorts. (C, D) The calibration curve of the nomogram in GSE84433 and GSE84437 cohorts. (E, F) The ROC curves at 1-, 3-, and 5-years of nomogram in GSE84433 and GSE84437 cohorts. AUC, areas under the curves.


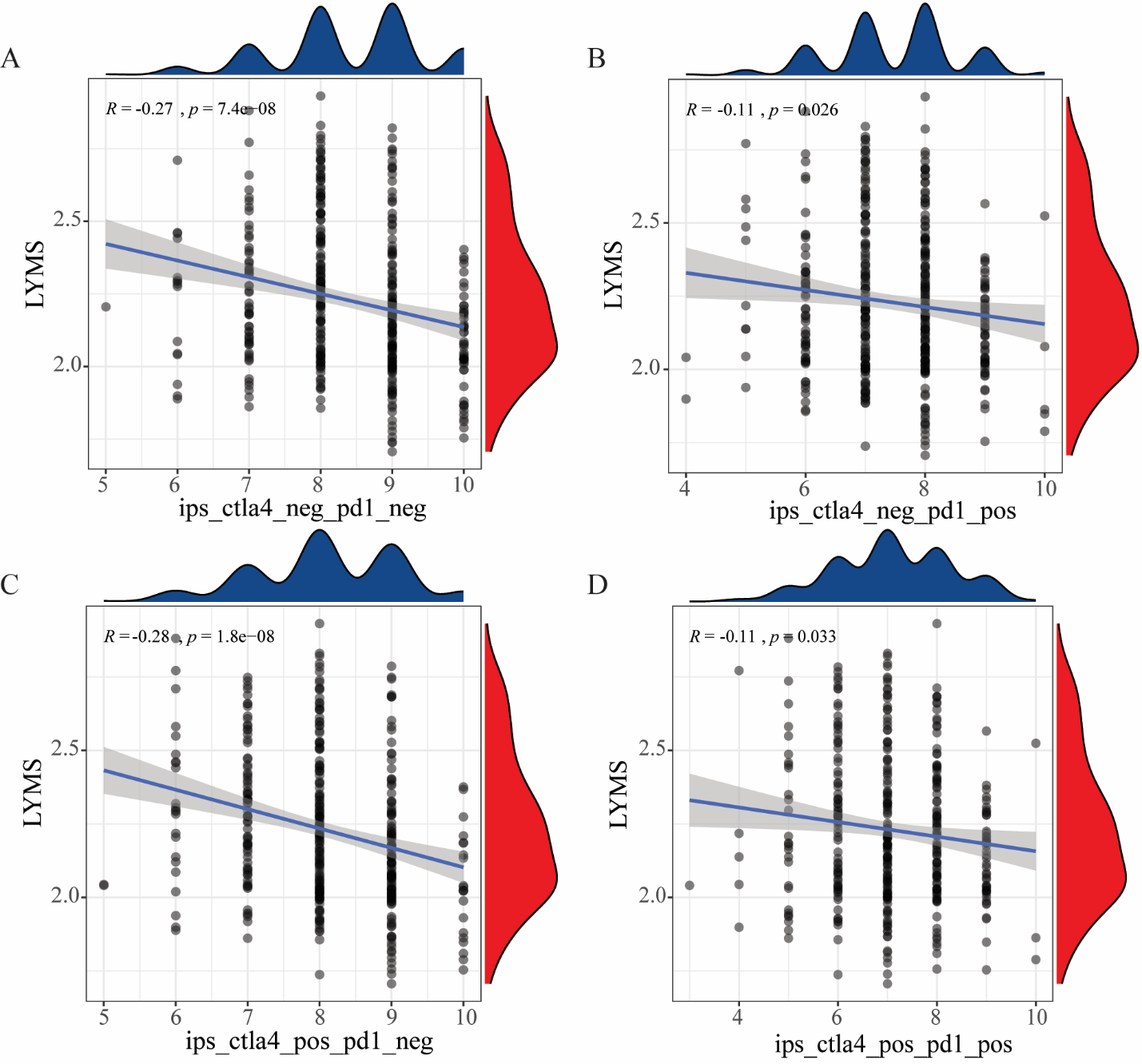


**Supplementary Figure 8. The correlation between the LYMS and IPS in various subgroups.** (A) CTLA4-negtive and PD1-negtive, (B) CTLA4-negtive and PD1-positive, (C) CTLA4-positive and PD1-negtive, (D) CTLA4- positive and PD1- positive.


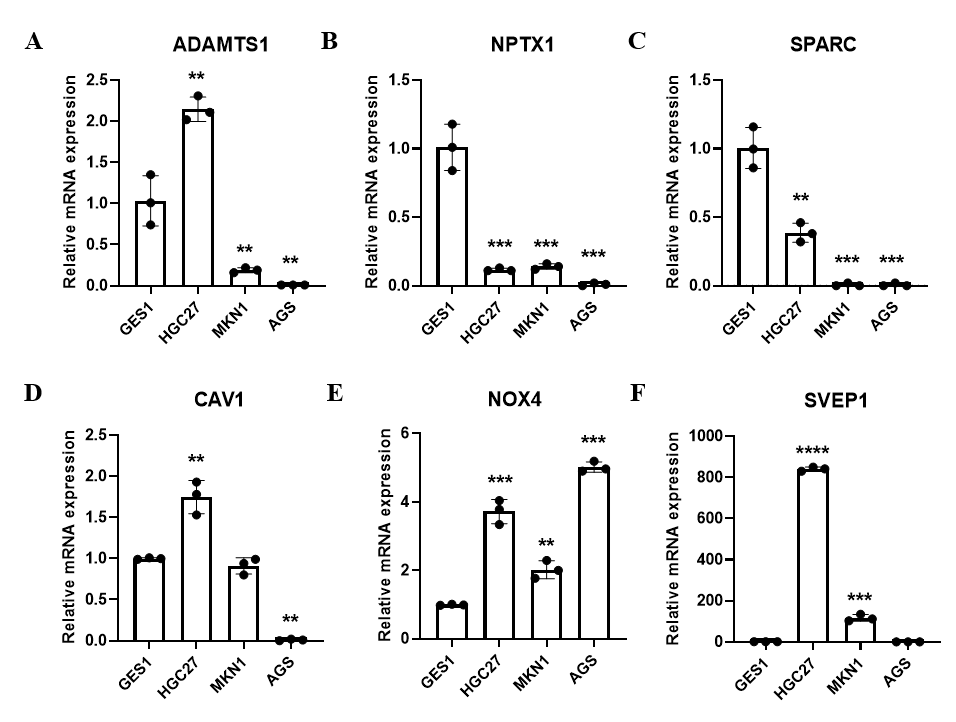


**Supplementary Figure 9. Validation of the expression of LYMS genes in various cell lines.** (A) ADAMTS1, (B) NPTX1, (C) SPARC, (D) CAV1, (E) NOX4, (F) SVEP1. n=3; **, P＜0.01; ***, P＜0.001; ****, P＜0.0001.
